# Pathogenesis of adolescent idiopathic scoliosis in girls - a double neuro-osseous theory involving disharmony between two nervous systems, somatic and autonomic expressed in the spine and trunk: possible dependency on sympathetic nervous system and hormones with implications for medical therapy

**DOI:** 10.1186/1748-7161-4-24

**Published:** 2009-10-31

**Authors:** R Geoffrey Burwell, Ranjit K Aujla, Michael P Grevitt, Peter H Dangerfield, Alan Moulton, Tabitha L Randell, Susan I Anderson

**Affiliations:** 1 Centre for Spinal Studies and Surgery, Nottingham University Hospitals Trust, Queen's Medical Centre Campus, Nottingham, UK; 2 School of Biomedical Sciences, University of Liverpool, Liverpool, UK; 3 Department of Orthopaedic Surgery, King's Mill Hospital, Mansfield, UK; 4 Department of Child Health, Nottingham University Hospitals Trust, Queen's Medical Centre Campus, Nottingham, UK; 5 School of Biomedical Sciences, University of Nottingham, Nottingham, UK

## Abstract

Anthropometric data from three groups of adolescent girls - preoperative adolescent idiopathic scoliosis (AIS), screened for scoliosis and normals were analysed by comparing skeletal data between higher and lower body mass index subsets. Unexpected findings for each of skeletal maturation, asymmetries and overgrowth are not explained by prevailing theories of AIS pathogenesis. A speculative pathogenetic theory for girls is formulated after surveying evidence including: (1) the thoracospinal concept for right thoracic AIS in girls; (2) the new neuroskeletal biology relating the sympathetic nervous system to bone formation/resorption and bone growth; (3) white adipose tissue storing triglycerides and the adiposity hormone leptin which functions as satiety hormone and sentinel of energy balance to the hypothalamus for long-term adiposity; and (4) central leptin resistance in obesity and possibly in healthy females. The new theory states that AIS in girls results from developmental disharmony expressed in spine and trunk between autonomic and somatic nervous systems. The autonomic component of this *double neuro-osseous theory *for AIS pathogenesis in girls involves *selectively increased sensitivity *of the hypothalamus to circulating leptin (genetically-determined up-regulation possibly involving inhibitory or sensitizing intracellular molecules, such as SOC3, PTP-1B and SH2B1 respectively), with asymmetry as an adverse response (hormesis); this asymmetry is routed bilaterally via the sympathetic nervous system to the growing axial skeleton where it may initiate the scoliosis deformity (leptin-hypothalamic-sympathetic nervous system concept = *LHS concept*). In some younger preoperative AIS girls, the hypothalamic up-regulation to circulating leptin also involves the somatotropic (growth hormone/IGF) axis which exaggerates the sympathetically-induced asymmetric skeletal effects and contributes to curve progression, a concept with therapeutic implications. In the somatic nervous system, dysfunction of a postural mechanism involving the CNS body schema fails to control, or may induce, the spinal deformity of AIS in girls (*escalator concept*). Biomechanical factors affecting ribs and/or vertebrae and spinal cord during growth may localize AIS to the thoracic spine and contribute to sagittal spinal shape alterations. The developmental disharmony in spine and trunk is compounded by any osteopenia, biomechanical spinal growth modulation, disc degeneration and platelet calmodulin dysfunction. Methods for testing the theory are outlined. Implications are discussed for neuroendocrine dysfunctions, osteopontin, sympathoactivation, medical therapy, Rett and Prader-Willi syndromes, infantile idiopathic scoliosis, and human evolution. AIS pathogenesis in girls is predicated on two putative normal mechanisms involved in trunk growth, each acquired in evolution and unique to humans.

## Introduction

The autonomic nervous system through its hypothalamic neuroendocrine control of puberty, menarche and skeletal growth [1-3] contributes importantly to the pathogenesis of AIS [4-6]. Melatonin [7-13] and its signaling pathway dysfunction [14-20] and platelet-calmodulin dysfunction [21,22] detected in AIS subjects involve the autonomic nervous system. In AIS girls, autonomic nervous system activity was reported to be higher than controls [23].

**Figure 1:**
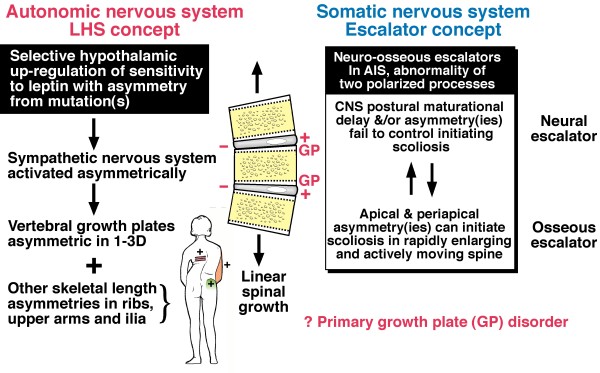
***Double neuro-osseous theory *for the pathogenesis of AIS in girls**. Disharmony in spine and trunk between the two nervous systems, autonomic (*leptin-hypothalamic-sympathetic nervous system - LHS - concept*) and somatic (*escalator concept*). The drawing of the girl shows three extraspinal sites where left-right skeletal length asymmetries have been detected in AIS subjects - ribs [30,31], upper arms [32] and iliac height [33,34]; the latter two asymmetries correlate significantly with adjacent spinal curve severity suggesting the presence of vertebral growth plate asymmetries [32,34-36]. Asymmetries are also found in tibial lengths [34,37], femoral anteversion [38,39], femoro-tibial correlations [40,41] but not tibial torsion [39,42]. There is some evidence suggesting a "primary" vertebral growth plate disorder in AIS [43,44] but this is controversial [45].

The *double neuro-osseous theory *for AIS pathogenesis in girls postulates developmental disharmony between somatic [24] and autonomic [25,26] nervous systems [27-29] expressed in the spine and trunk and exaggerated by hormones producing systemic skeletal overgrowth (preoperative girls) (Figure 1) [30-45]. The theory predicates AIS pathogenesis in girls on dysfunction in one or both of two putative *normal *mechanisms involved in trunk growth, each acquired in evolution and unique to humans, namely:

*(1) Physiological trunk width skeletal growth *driven hormonally and supplemented by the sympathetic nervous system acting symmetrically [25,26,46-50].

*(2) Physiological trunk postural mechanisms of the somatic nervous system *adapting normally to the growing and biomechanically changing skeletal framework [24,51,52].

There is preliminary evidence suggesting that the hypothalamus of some normal juvenile girls, but not boys, functions with *central leptin resistance of the somatotropic (growth hormone/IGF) axis*. This mechanism may limit the energy invested in female skeletal growth thereby conserving energy for reproductive development [50]. AIS in girls is viewed here as commonly resulting from increased central leptin *sensitivity *of hypothalamic sympathetic functions and, in some girls, of the somatotropic (growth hormone/IGF) neuroendocrine axis.

These concepts provide an evolutionary and biological perspective [53] of energy homeostasis (bioenergetics) [54], particularly involving white adipose tissue storing excess energy as triglycerides, from which the double neuro-osseous theory is formulated. At the molecular level, disharmony between genes is established [55]. Gene variants that may impact the biology of AIS pathogenesis [56] are considered here in relation to body mass index (BMI), timing of puberty, leptin, leptin-receptor deficiency, changes in hypothalamic resistance/sensitivity to leptin, some hormones thought to be related to AIS pathogenesis, and certain genetically-modified mice.

The *double neuro-osseous theory *accommodates evidence that AIS may not be a single condition [51,57-65]. This it explains by different relative contributions to the trunk deformity by the autonomic (sympathetic nervous system and hormone effects) and somatic nervous systems (postural mechanisms), which can vary between subjects.

The aims of this paper are to:

• outline some anthropometric findings for AIS girls not explained by prevailing theories of pathogenesis;

• provide a novel theoretical framework for AIS pathogenesis in girls to explain the findings and connect knowledge from several biological fields;

• suggest tests of the theory including endocrine studies;

• focus on therapeutic implications and some possible manipulatable causes;

• consider an evolutionary perspective [53] for the pathogenesis of AIS in girls stemming from female fat accumulation in puberty; and

• foster new thinking and research to improve causal knowledge of AIS pathogenesis.

## Background

### General comments

Most experts agree that the causes of adolescent idiopathic scoliosis (AIS) are multifactorial with no generally accepted theory of pathogenesis (Appendix 1) [14,51,57,58,66-111]. This reflects shortcomings in our understanding of the complex biological and biomechanical multifactorial processes involved in AIS pathogenesis which needs innovative thinking [73], to which we add new findings not explained by prevailing theories. One recent review suggests that genetics and the unique mechanics of the fully upright human spine play a decisive role in AIS pathogenesis [75]. A genome-wide association study revealed 30 markers identified as the most useful prognostically [56].

### Biomechanical spinal growth modulation

A commonly held pathogenetic theory is that initiating changes in the spine of unknown origin lead to biomechanical spinal growth modulation causing curve progression [80-82,107]. Brace treatment is based on this view of pathogenesis.

### Neurological abnormalities

Studies over many years in AIS subjects have shown abnormalities of visual, vestibular, proprioceptive and postural control [67,69,70,94-96,99-104] involving the brain stem [69,95,97-99], cerebral hemispheres and corpus callosum [69,95,104,111-115], though not without controversy. Lowe et al [67] suggested that the pathogenesis of adolescent idiopathic scoliosis (AIS) results from a primary pathology in the hind brain causing a defect of central control, or processing in the central nervous system (CNS) that affects a normal growing spine [116]. Neurological abnormalities with AIS have been explained by four fairly comprehensive concepts for pathogenesis:

(1) *visuo-spatial perceptual impairment producing a motor control problem *[104];

(2) *body-spatial orientation concept *[69];

(3) *neurodevelopmental concept *[105,106]; and

(4) *sensory integration disorder *[102].

Abnormal asymmetries of brain structure and function are found in AIS girls for each of cerebral hemispheres [112-115], dichotic listening [112], brain stem [97-99] and, in preliminary research for left thoracic AIS, on MR brain scans, reduced white matter density in the left internal capsule and corpus callosum [114,115].

### Origins of the double neuro-osseous theory - the escalator concept

**Figure 2:**
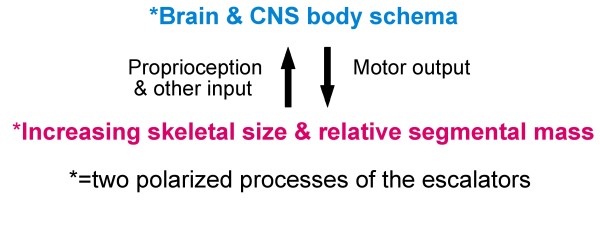
**Normality**. Osseous escalator and neural escalator (brain and CNS body schema) [24,51].

**Figure 3:**
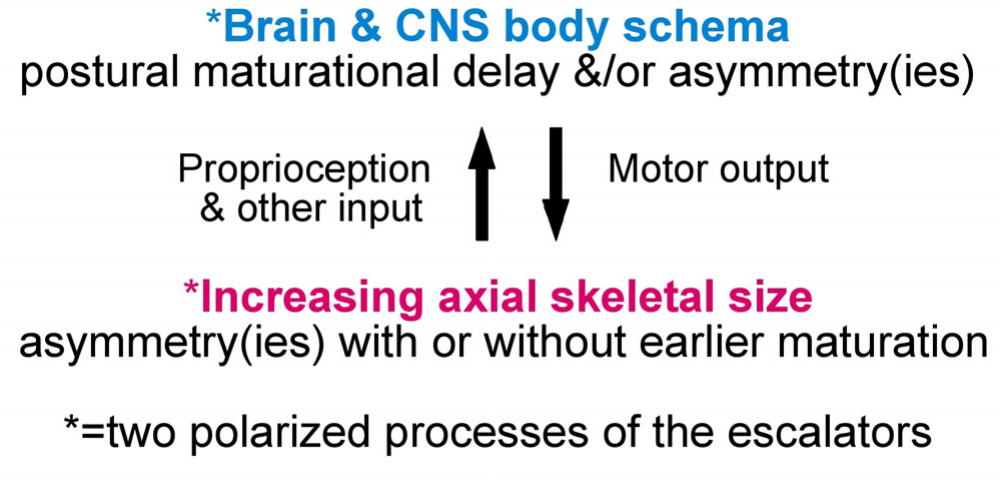
**AIS pathogenesis**. Abnormal neuro-osseous escalators as applied to the spine [24,51].

Summarizing concepts of AIS pathogenesis in 2008 [51], we suggested a novel *neuro-osseous escalator concept *for AIS in girls (Figures 1, 2 and 3). This involves interaction between the growing skeleton and postural mechanisms of the maturing somatic nervous system. The dependence of AIS progression on growth is attributed not to growth\velocity, but to rapid skeletal enlargement hormonally-induced, *producing skeletal sizes for age *beyond the capacity of postural mechanisms of the *somatic nervous system *to control the initiating deformity.

### Origins of the double neuro-osseous theory - the LHS concept

Later in 2008, from analyses of anthropometric data of adolescent girls - normal, screened and preoperative, we reported that relatively higher and lower subsets of body mass index (BMI) reveal different features of skeletal maturation [46,47,117-119] and asymmeties of spinal deformity and upper arm lengths [46,120,121] (Figure 1). Subsequently, skeletal overgrowth patterns for age were found in preoperative AIS girls compared with normal girls when analysed separately by higher and lower BMI subsets [29,122]. Then, in normal girls and boys, an excess of severe back humps was found to be associated with *lower BMI subsets *[123-125]. These and other findings were not explained by any of the theories surveyed (Appendix 1, items 1-15). A more comprehensive hypothesis for AIS pathogenesis in girls was needed incorporating energy homeostasis (bioenergetics) and the hypothalamus in a disorder presenting as abnormalities of trunk growth with axial and appendicular skeletal asymmetries and systemic skeletal features in preoperative girls. The components included in the new formulation are *white adipose tissue, leptin*, *hypothalamus *and *sympathetic nervous system *(*LHS concep*t). Together with the *escalator concept*, they form the *double neuro-osseous the*ory (Figure 1). It has common ground with the *thoracospinal concept *[59-63]. These findings for AIS girls and the severe trunk asymmetry of healthy adolescents [123-125] are consistent with the hypothesis that the control mechanisms of bioenergetics have relevance to the etiopathogenesis of such shape deformities/distortions.

## Scientific Basis of the Escalator Concept

### The central nervous system and the changes of the human frame during development and growth

Sporns and Edelman [126] wrote:

"*There is overwhelming evidence that the emergence of coordinated movements is intimately tied to both the growth of musculoskeletal system and to the development of brain. The neural development and learning cannot be considered outside of their biomechanical context. A key theoretical issue is how the changes in brain circuitry controlling muscles and joints become matched to simultaneously occurring developmental changes at the periphery."*

### CNS body schema ('body-in-the-brain')

The CNS body schema in adults is defined as a *".....system of sensory-motor processes that continually regulate posture and movement - processes that function without reflective awareness or the necessity of perpetual monitoring." *[127]. This control involves the posterior parietal cortex which participates in the dynamic representation of the *body schema *integrated with other cortical areas [127-130].

## SOMATIC NERVOUS SYSTEM - the escalator concept

### Normal adolescent girls

We postulate that during normal growth and maturation, a physiological balance is continuously renewed between two synchronous polarized processes we term *neuro-osseous timing of maturation (NOTOM) escalators *(Figure 2) [24,51,111], namely:

(1) *Osseous escalator*. Increasing skeletal size, changing skeletal shape and relative mass of the different body segments which, through posture and motion of the body by producing developmental biomechanical and kinematic changes at the periphery, create developmentally-altering proprioceptive and visuo-spatial inputs to the neural escalator in the brain.

(2) *Neural escalator and postural control*. The brain and *CNS body schema *are recalibrated as they continuously adjust to skeletal enlargement, shape and relative mass changes to enable them to coordinate motor actions. The *posterior parietal cortex (Area 7) *in human clinical and experimental studies has been shown to participate in the dynamic representation of the *CNS body schema *(Figure 2) [127-130]. Leptin functionally enhances NMDA receptors which are critically involved in most models of learning and memory [131,132]. Increased circulating leptin levels may explain the reduced grey matter of certain brain areas in obese subjects [133].

The term *escalators *are applicable only during growth. Muscles are not included in this terminology because they do not primarily drive skeletal growth, but have key roles in sensory and motor function and contribute to segmental masses. Similar mechanisms are being evaluated in robotics and specifically the learning in, and from, brain-based devices [134].

### Girls with AIS

Figures 1 and 3 provide an outline of the *escalator concept *for AIS pathogenesis in girls. Putative abnormalities of the two polarized components of the *escalators *- with asynchrony and asymmetry(ies) - provide the mechanisms of the *escalator concept *for AIS pathogenesis before and during the curve acceleration phase [5] in:

(1) *spine *growing rapidly with asymmetry(ies), and

(2) *brain and CNS body schema with -*

a) *postural maturational delay*, and/or

b) brain asymmetry(ies) [113-115].

Postural maturational delay in the *CNS *may be *relative to earlier skeletal maturation *[135-141], or *absolute *arising from an abnormality in afferent [100-103,142-145], central [104,113], or motor mechanisms [104,146]. A study of stroke subjects suggests that in axial postural control, the right hemisphere undertakes higher-order spatial processing than the left hemisphere [[147], see [148]].

The fate of early AIS - to progress, become static or resolve (rarely) according to the *double neuro-osseous theory *generally depends on the relative contribution and outcome of the disharmony (Figure 1) between:

a) vertebral growth plate asymmetries in up to three dimensions arising wholly or in part from dysfunction in the autonomic nervous system [25-29];

b) postural control, with or without asymmetries, of a rapidly enlarging and actively moving [52,71,149] adolescent spine; and

c) postural maturity (see Discussion, *Explanations for undisputed facts about AIS*, (2) *Predilection for females b)*).

### Postural scoliosis in melatonin-deficient mice

*Bipedal mice and the protection by melatoni*n. Machida et al [150] suggested that the scoliosis development in bipedal melatonin-deficient mice and the protection from scoliosis by restoring melatonin levels, are crucial influences for a postural mechanism and bipedalism in scoliosis development. Deficiency of osteopontin or CD44 receptor also protect transgenic melatonin-deficient C57Bl/6J mice from scoliosis [19,20]. Later, we examine whether the scoliosis of these three mouse models may be markers of stress reactions involving the hypothalamus rather than crucial influences for scoliosis development (see Scientific basis of leptin-hypothalamic-sympathetic nervous system (*LHS*) concept, items 11 & 12).

## Some Observations on Skeletal Maturation Relating to AIS not Explained by Pathogenetic Theories (Appendix 1, Items 1-15)

### Prescoliotics and early skeletal maturation of AIS subjects

Little discussed features of AIS pathogenesis are:

• Prescoliotics of both sexes show body height, sitting height, and growth of sitting height greater than in non-scoliotic children [135,136].

• Early radiological maturation at 11-12 years of age in AIS subjects [137].

• Early adolescent skeletal growth attained for age by AIS girls [38,39,41,121,135-141]. In the preoperative AIS girls of the relatively higher BMI subset, all the skeletal parameters we measured when plotted as standard deviation scores against age, showed negative regressions - several statistically significant, but not for the lower BMI subset of preoperative AIS girls (unpublished observations).

Together, these observations suggest that, collectively, AIS girls have a growth pattern different from normal, involving growth factors connected to the disease [137,151], confirmed in subsequent research [64,65,89,90,152].

### Extra-spinal skeletal length asymmetries detected with AIS

Periapical ribs longer on the concavity of right thoracic AIS in elderly scoliosis cadavers were found [30] and given pathogenetic significance, but the finding is controversial [31,63]. In thoracic idiopathic scoliosis, upper arm length asymmetry (relatively longer on convexity) is significantly associated with each of apical vertebral rotation (AVR) and Cobb angle [32]. Also in scoliosis subjects but with lower spine scoliosis (thoracolumbar and lumbar), iliac height asymmetry (relatively taller on concavity) is associated with Cobb angle and apical vertebral rotation [34], confirming an observation for subjects with lumbar scoliosis [33].

It is unknown whether these asymmetries of upper arm, iliac height and also femoral anteversion [38,39] are pathogenetically-related to any local asymmetry in the AIS spine. We speculate that they are [24,25,32,34-37,40,41,105,106,120,121]. In this connection we outlined evidence supporting a common pathogenesis of upper arm length asymmetry and thoracic AIS spinal deformity [32]. In a similar way that the extraspinal general skeletal overgrowth for age in AIS girls is associated with the relative anterior spinal overgrowth (RASO) [64,65,89,90] giving it pathogenetic significance, we view the abnormal asymmetry of paired bones as *sentinels *of vertebral and/or rib growth plate asymmetries and having pathogenetic significance. There is some evidence of a primary vertebral growth plate disorder in AIS (Figure 1) [43,44,65,90]. Extra-spinal skeletal length asymmetry is also found in ilio-femoral lengths [35]. More such asymmetries need to be sought in other bilateral bones of AIS girls - sacral alae [153-155], clavicles and scapulae.

## Body Mass Index (BMI) Relating to AIS and Causal Genes

BMI is usually expressed as weight in kg/height in m^2^. Standards are available for the UK in *The 'Healthy Living' Social Market Initiative *[156]. BMI does not distinguish between fat and muscle mass. The balance between energy intake and output determining BMI is largely controlled by powerful unconscious mechanisms within the autonomic nervous system (see Scientific basis of leptin-hypothalamic-sympathetic nervous system (*LHS*) concept, item 3).

### BMI and AIS

In girls with AIS and young adults with scoliosis, lower body mass index [157-165] has been found by most but not by all workers [46,135,166,167] These findings have implications for body development, abnormal spinal development, or nutrition of patients with AIS [165]. There is some evidence of disordered eating behavior [159,168,169], but the low body-mass index of girls with AIS is said not to be the result of the eating disorder [168].

### Overweight AIS patients

There is a trend towards increasing numbers of adolescents with AIS in the overweight category [170,171]. The hypothesis that increased BMI can influence scoliosis presentation was tested in 427 adolescents with idiopathic scoliosis [170]. Female subjects who presented with larger curves (>50 degrees) were older and had a greater BMI than those with curves less than 50 degrees (p = 0.0557). Possible curve detection difficulties, endocrine factors and an earlier puberty with increased fat mass were suggested for the association of the larger curves with obesity.

### Fat mass related to bone mass and genetic markers in normal children

In humans, common variants at only two loci, *FTO *and *MC4R *(melanocortin-4 receptor) have been reproducibly associated with body mass index (BMI) [172,173]. Mutations of *MC4R *are the leading cause of severe childhood-onset obesity [172]. A meta-analysis of 15 genome-wide association studies for BMI identified six additional loci, including *SH2B1 *[173]. Several of the likely causal genes are expressed, or known to act in the central nervous system [172-174]. Different versions of the human gene *FTO *strongly correlate with BMI [174]; the *FTO *gene with significant polymorphic variation has been identified in several papers as a candidate gene predisposing to obesity. In rats, *fto *is significantly up-regulated (41%) after food deprivation [174]. In humans, fat mass, and genetic markers for obesity genes *MC4R *and *FTO*, are strongly related to bone mineral content, total body and regional, measured by DXA [175].

*SH2B1 *is a strong prior candidate for regulating body weight; it is implicated in leptin signaling; *Sh2b1*-null mice are obese; and the evidence suggests that the effects of this gene on obesity are mediated through the central nervous system [173] (see *Leptin, hypothalamus and AIS*).

Overall, these findings support the view that fat mass is on the causal pathway for bone mass in normal children [175].

### Fto gene in mice

In mice, loss of the *Fto *gene leads to postnatal growth retardation, reduction of adipose tissue and serum leptin, increased energy expenditure, enhanced circulating levels of adrenaline and noradrenaline; these changes are attributed to sympathetic system activation (sympathoactivation) controlling energy expenditure through mitochondria and fatty acids/triglycerides [176,177]. In *Fto*-deficient mice the sympathoactivation associated with decreased circulating leptin levels is similar to the hypothalamic up-regulation and sympathoactivation we postulate for AIS girls, but without the skeletal overgrowth for age (see Autonomic nervous system - leptin-hypothalamic-sympathetic nervous system (*LHS*)-driven mechanism in health and *LHS concept *in AIS).

### Relation of relatively higher and lower BMIs to skeletal sizes and asymmetries in AIS girls

Most previous research on AIS has evaluated BMI as a sole parameter, or in relation to a few skeletal features [163,164,167]. The genetic aspects of BMI for AIS have not been reported but it may be difficult in such research to disentangle the contributions of lower BMI from that of the AIS.

Our recent findings for AIS girls show that *higher and lower BMI subsets relative to median BMI values for ag*e have different patterns by each of (1) skeletal sizes for age, (2) bilateral skeletal length asymmetries, and (3) skeletal overgrowth for age in preoperative AIS compared with normal girls, which is systemically distributed suggesting hormonal effects.

## Body Mass Index (BMI) Subsets in AIS and Normal Girls Reveal Effects of Energy Stores on Skeletal Maturation, Asymmetry and Overgrowth: Summary of Recent Findings

Three groups of adolescent girls were measured: normals (n = 274 in 1973-81); routinely screened for scoliosis using a prescribed method [178] (n = 137 in 1988-2001); and preoperative (n = 122 in 1992-99). The possibility that observed skeletal differences were due to secular changes (except for sitting height at 10 years of age) was excluded by comparing data from healthy girls measured in 1994-6 with those measured in 1973-81 [140]. The BMIs were not significantly different between groups with 4.7%, 4.6% and 5.6% respectively outside the 95% confidence intervals of the BMI values, almost entirely overweight. These percentages are lower than expected from societal changes [156].

**Figure 4:**
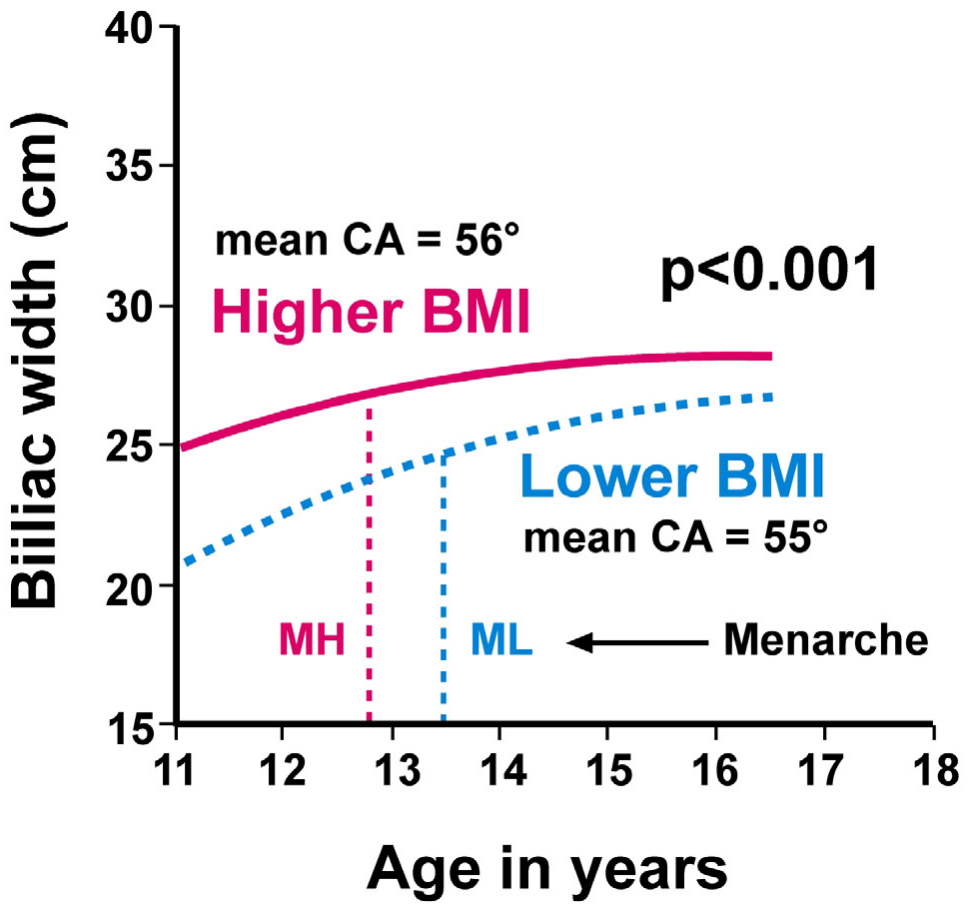
**Biiliac widths for preoperative girls**. Graphs showing best-fit quadratic regression lines by age in years for higher (n = 65) and lower (n = 57) BMI subsets relative to median BMI values by each year of age (CA = Cobb angle, mean BMIs 21.7 and 17.3 respectively, p < 0.001). The girls in the higher BMI subset have larger biiliac widths for age relative to those in the lower BMI subset (p < 0.001, correcting for menarcheal age p = 0.020). Mean menarcheal ages for relatively higher (MH) and lower BMI (ML) subsets are 12.82 years and 13.43 years (p = 0.048, premenarcheal n = 7 & 19 respectively)(analyses of variance correcting for age or menarcheal age).

**Figure 5:**
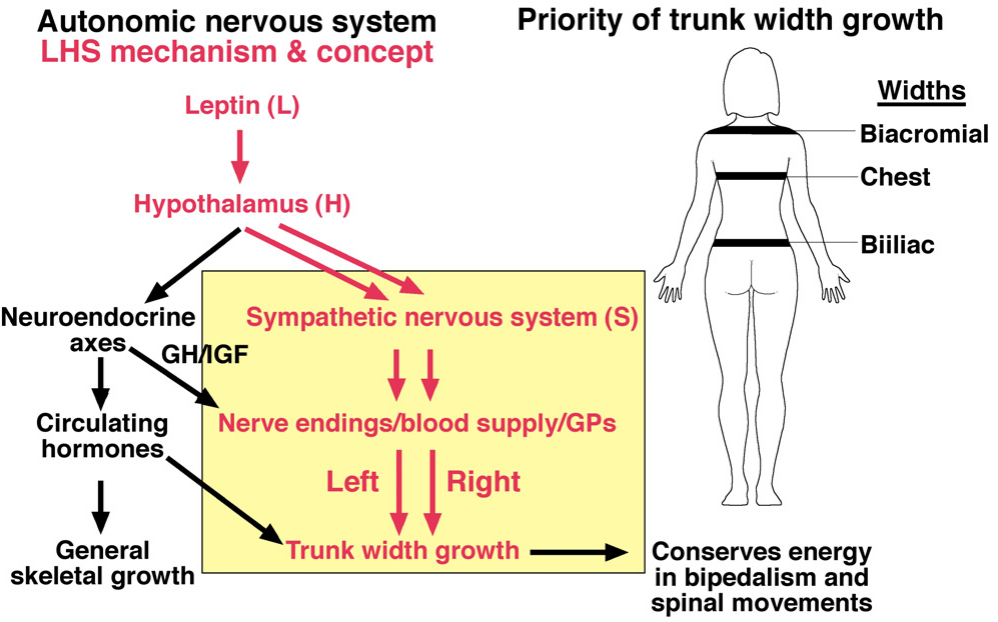
**In the autonomic nervous system of normal adolescent girls, the *leptin-hypothalamic-sympathetic nervous system (LHS)-driven mechanism (red) *supplements bilaterally the blood-borne hormonal contribution (lowest oblique arrow) to trunk width growth at the pelvis, chest and shoulders (yellow box) with little or no sympathetic nervous system (SNS)-induced effect in the limbs (upper arms, forearm-with-hands, tibiae and feet) **[46,117-119]. In the preoperative AIS girls, the *LHS concept *suggests that the GH/IGF axis (upper arrow labeled GH/IGF) and possibly estrogen [122], causes exaggeration of the SNS-induced vertebral/rib length asymmetry with both GH/IGF and sympathoactivation contributing to scoliosis curve progression (Figure 6) in an inverse pathogenetic relationship. The *LHS concept *suggests that both putative mechanisms, GH/IGF and SNS, provide therapeutic potential for progressive AIS in girls (GPs = growth plates, see Endocrine and Therapeutic Implications).

*Energy priority of trunk width growth is revealed by body mass index (BMI) subsets in adolescent girls (Figures *4* and *5*) - intrinsic or extrinsic mechanisms? A contrast with vertebral length growth in melatonin-deficient mice*

Figure 4 shows that preoperative girls in the higher BMI subset have larger biiliac widths for age relative to those in the lower BMI subset (p < 0.001). We reported that *BMIs above and below mean (now median) levels *separated girls with relatively earlier and larger trunk width at each of the pelvis, chest and shoulder girdle for each of a) preoperative, b) screened [46,117-119] (except for biacromial width in screened girls), c) normal adolescent girls [47,48], and d) normal juvenile girls at 5-10 years [49] with little or no such effect in limb segment lengths (Figure 5). We term this phenomenon *energy priority of trunk width growth*. Normal boys show this BMI effect on skeletal maturation in trunk widths and, unlike girls, also in the limbs during adolescence [47,48] and at 5-10 years [49].

• *"Energy"*, is used because relatively higher BMI probably implies relatively higher circulating leptin indicating more energy available from fat.

• *"Priority"*, is used because growth plates (GPs) contributing to the trunk width of girls, take priority over those in limbs in "tapping" available energy.

(1) How does the higher BMI subset of preoperative girls attain greater biiliac width for age than the lower BMI subset? The earlier menarcheal age of the higher BMI subset with earlier puberty suggests hormonal effects cause earlier iliac maturation with relative overgrowth of younger AIS girls.

(2) Why is this BMI-related earlier maturation of trunk widths - biiliac, chest and biacromial in girls scarcely found in the limb lengths of girls? (Figure 5). The growth plates in trunk and limbs may respond -

• *intrinsically *and differently to hormones by genetic programs established in early embryogenesis, and/or

• *extrinsically *in the presence of any sympathetic nervous system innervation (see Autonomic nervous system - leptin-hypothalamic-sympathetic nervous system (*LHS*)-driven mechanism in health and LHS concept in AIS).

### Energy priority of trunk length growth in leptin-deficient mice?

In leptin-deficient mice (*ob/ob*) altered leptin signaling has significantly different effects on bone growth in the axial and appendicular skeletons [179]. Compared with normal mice, leptin-deficient mice have significantly shorter femora, and significantly increased vertebral lengths, a trend confirmed in subsequent research [180]. Suggested reasons for this axial/appendicular skeletal growth difference in mice include: (1) decreased thigh muscle mass as a factor for the femoral shortening through mechanotransduction pathways [179]; and (2) vertebral growth plates respond to absent leptin signals in a fundamentally different manner from long bone growth plates [180]. The latter interpretation is consistent with the view that leptin-deficient mice have energy priority of *vertebral linear growth *relative to limb bones, in contrast to the energy priority of *trunk width growth *in girls (Figure 4). This apparent human/mouse difference is consistent with an evolutionary change to the trunk broadening of hominins (Figure 5) (hominins include living humans and fossil species that are ancestral to living humans, see Evolutionary Origins).

#### Skeletal asymmetries

### Mean upper arm length asymmetries in preoperative girls

In the *lower BMI subset*, mean upper arm length asymmetry (7.0 mm, right *minus *left)) is significantly greater preoperative than in screened (-0.8 mm) and normal girls (2.1 mm)(each p < 0.001 with statistically significant variance ratios). In the *higher BMI subset*, mean upper arm length asymmetries are respectively 3.7 mm, 1.1 mm, and 2.4 mm, greater in preoperative than screened girls (p = 0.031) (analyses of variance correcting for age) [46].

### Right thoracic AIS, curve severity and upper arm length asymmetries

**Figure 6:**
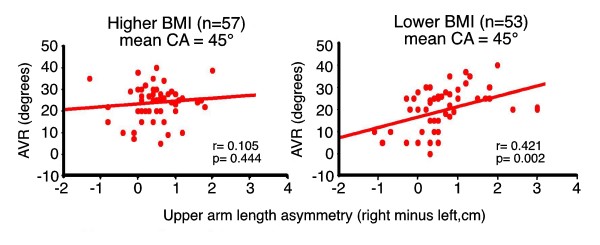
**Right thoracic AIS girls from preoperative (n = 77) and screened (n = 33) girls**. Linear regression analyses, Pearson correlation coefficients and scatter diagrams of apical axial vertebral rotation (AVR, Perdriolle [181]) against upper arm length asymmetries (right minus left) for higher (n-57) and lower (n = 53) BMI subsets (mean BMIs 21.8 and 17.3 respectively, p < 0.001). Note, the statistically significant correlation for the lower (p = 0.002, r = 0.421) but not higher BMI subset (p = 0.444, r = 0.105); the difference between higher and lower BMI subsets after correcting for menarcheal age is statistically significant for AVR (p = 0.001) but not Cobb angle (p = 0.199). Mean Cobb angles 45.4/45.4 degrees of similar curve types; mean AVRs 23.9/19.7 degrees (p = 0.015) both independent of age; mean upper am length asymmeties (right *minus *left) 4.7/6.7 mm (p = 0.172) both significantly different from normals (p = 0.005/p < 0.001); mean menarcheal ages 12.69 years and 13.31 years (p = 0.046, premenarcheal n = 5 & 14 respectively) (ANOVAs correcting for age) [46,120,121].

Figure 6[181] shows that apical vertebral rotation is significantly associated with upper arm length asymmetry for the *lower*, but not *higher BMI subset*, also for Cobb angle (p < 0.001, r = 0.510) [46,120,121]. These findings suggest that the abnormal upper arm length asymmetry of thoracic AIS [32] is not secondary to the spinal deformity but has a pathogenesis common to the spinal deformity [32].

### Right thoracic AIS, upper arm length asymmetry and age

**Figure 7:**
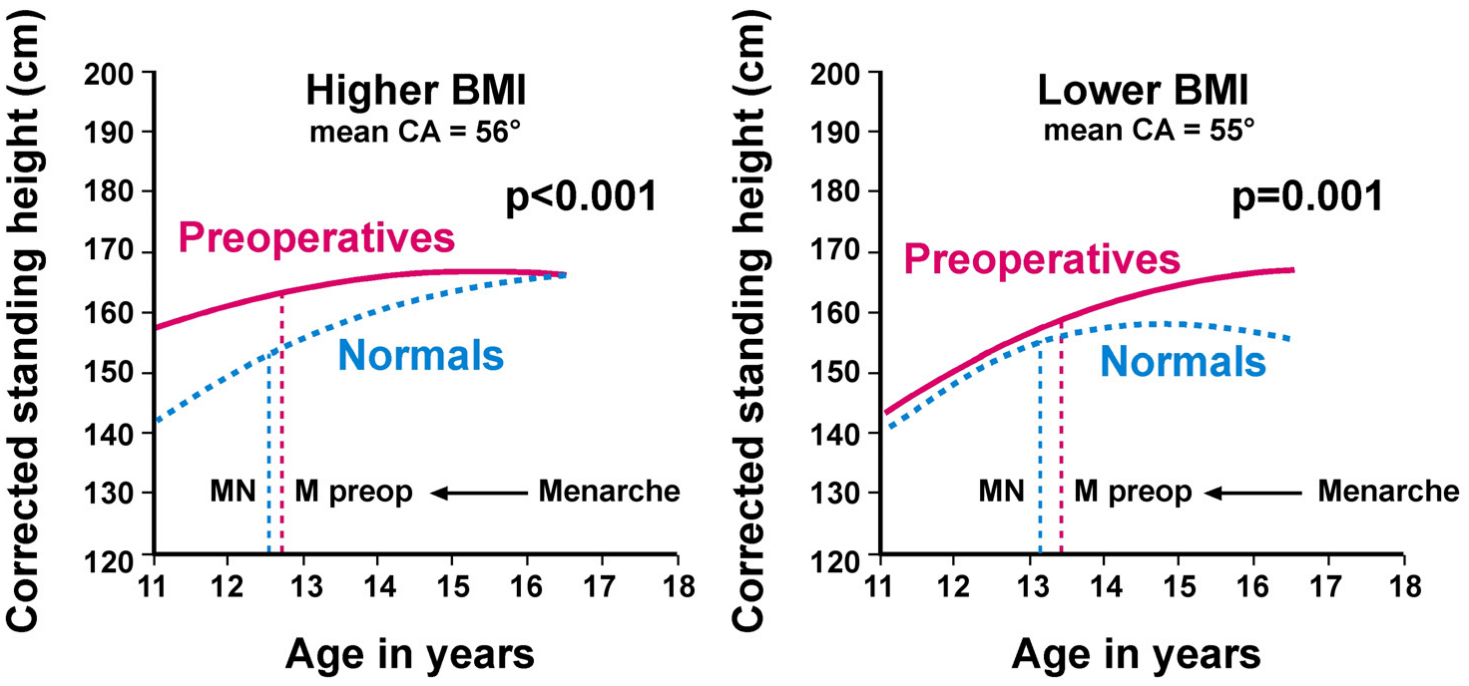
**Corrected stature by age for preoperative and normal girls**. Corrected standing height (by the Bjure-Nachemson formula [182]) plotted against age in years for relatively higher (n = 65) and lower (n = 57) BMI subsets (CA = Cobb angle). Graphs show best-fit quadratic regression lines for preoperative and normal girls with p values for differences between preoperatives and normals (correcting for menarcheal age p < 0.001 for each BMI subset). MN = menarcheal age of normals, M preop = menarcheal age of preoperative girls: mean menarcheal ages of preoperatives and normals in *higher BMI subset *12.82 years and 12.59 years (p = 0.717); and *lower BMI subset *13.43 years and 13.14 years (p = 0.825, premenarcheal for normals n = 45 & 63 respectively). Mean BMIs for preoperatives as in Figure 4, and for normals 21.0 (n = 139) and 17.3 (p < 0.001 n = 135) (ANOVAs correcting for age or menarcheal age) [29,122].

In girls with right thoracic AIS, mean upper arm length asymmetry is significantly greater than normal girls (5.6/2.2. mm, p < 0.001). The asymmetry is similar at 11-12 years of age in both higher and lower subsets. It negatively regresses on age in *the higher BMI subset *(p < 0.001, r = -0.486) but not significantly in the *lower BMI subset *(p = 0.125, r = -0.212, variance ratio of lower to higher BMI subset = 2.05, p < 0.01); and menarcheal age negatively regresses on upper arm length asymmetry in *the higher BMI subset *(p = 0.027, r = -0.325). This 'transient' asynchronous upper arm length growth detected with abnormal systemic earlier skeletal overgrowth for age as in some younger preoperative girls (Figure 7), suggests a relation to pathogenesis. There were insufficient girls with left thoracic AIS for separate analyses (n = 12 [46]) (see Discussion, *Upper arm length asymmetry and the higher BMI subset of right thoracic AIS, and Skeletal asymmetries and lower BMI subsets*).

### Skeletal overgrowth for age in preoperative AIS/normal girls (Figure 7)

Figure 7[182] shows that with relatively *higher BM*Is, the younger AIS girls, have larger corrected stature for age than do the normal girls, becoming normal sizes by 16 years of age (p < 0.001, ANOVA with age correction). This pattern is found in each of 11 skeletal segments, four of them in bilateral limb segments suggesting a systemic response. Mean menarcheal ages are not significantly different. The skeletal pattern for age suggests earlier skeletal maturation with overgrowth in these younger girls probably from circulating hormones ? GH/IGF-I and possibly estrogen [29,122]. The AIS girls with *relatively lower BMIs *show a more complex pattern with two growth phases: earlier phase similar to normals, and later phase in most skeletal segments, mainly postmenarcheal, with larger overall skeletal growth attained for age in preoperatives relative to normals, ? estrogen effect [29,122]. The similar mean Cobb angle and apical vertebral rotation show that while curve severity at the time of surgery appears independent from (1) skeletal growth patterns, and (2) BMI subsets, we suggest that common factors in different proportions and other common factors, determine the similar curve severities in both subsets (see Discussion *Skeletal sizes for age - curve severity, sympathoactivation and hormonal stimulation*).

### Back contour asymmetry in normal girls and boys

The excess of severe back humps in girls and boys was associated with *lower BMI subsets *[123-125].

### Considered together, the above findings are not explained by any of the prevailing theories of AIS pathogenesis (Appendix 1, items 1-15)

A more comprehensive hypothesis for girls with AIS was required involving energy homeostasis and the hypothalamus in a disorder presenting as abnormalities of trunk growth with axial and appendicular skeletal asymmetries and in preoperative girls with systemic skeletal features.

## Scientific Basis of Leptin-Hypothalamic-Sympathetic Nervous System (*LHS*) Concept

From a novel interpretation of the above findings, the leptin-hypothalamic-sympathetic nervous system (*LHS*) concept for AIS pathogenesis was formulated [25,26] after surveying evidence relating to:

1. Thoracospinal concept.

2. New neuroskeletal biology.

3. Energy homeostasis and sympathetic nervous system.

4. White adipose tissue, *leptin*, hypothalamus, sympathetic nervous system and bone formation/resorption in health.

5. Leptin and bone growth in mice.

6. Leptin and bone growth in children.

7. Leptin, hypothalamus and AIS.

8. Central leptin resistance in obesity and possibly in healthy females.

9. AIS as a systemic disorder - platelet calmodulin dysfunction.

10. AIS as a systemic disorder - melatonin, melatonin signaling, osteopontin and soluble CD44 receptor.

11. Some melatonin-deficient mouse models of scoliosis - markers of developmental stress?

12. Osteopontin and bone remodeling in mice.

13. Melatonin receptor 1B (MT1B), AIS, glucose metabolism and type 2 diabetes.

### Thoraco-spinal concept

**Figure 8:**
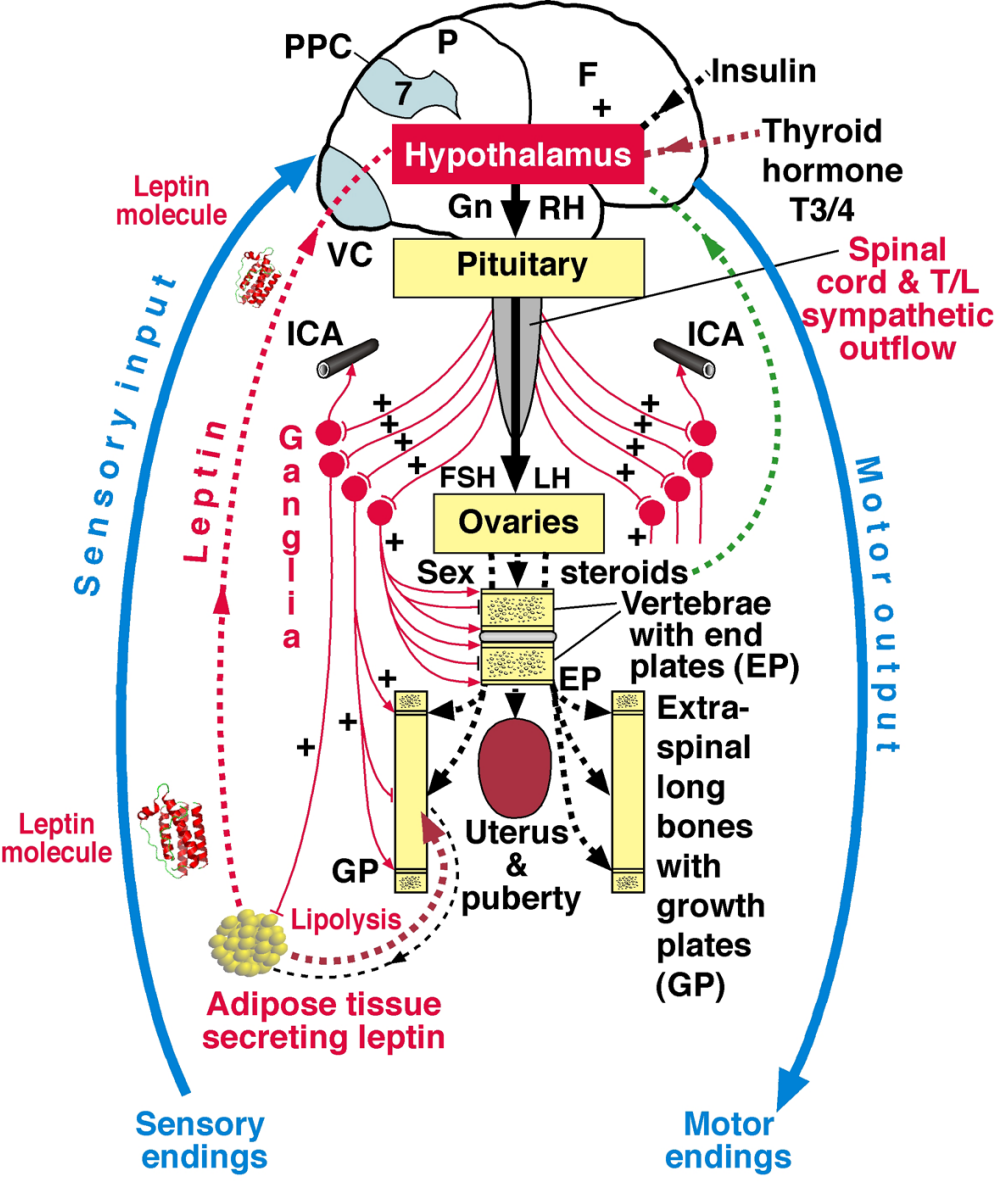
**Diagram of relevant somatic (blue) and autonomic nervous systems (ANS, red) in girls**. Note, *leptin, hypothalamic-pituitary-ovarian axis *and *sympathetic axis *in the ANS. The sensory input, motor output and PPC relate to the *somatic nervous system *and the rest illustrate *leptin, hypothalamus and sympathetic nervous system of the LHS mechanism and concept*. The neuroendocrine control of the female reproductive axis and the bilateral sympathetic nervous system control of skeletal and adipose tissues are shown. Ganglia = ganglionated sympathetic trunk. Sympathetic nerves are shown as thin continuous lines and hormones as interrupted lines. Pre- and post-ganglionic sympathetic nerves are shown bilaterally with arrows indicating enhancement of function, and blunted lines as inhibition. F = frontal lobe, P = parietal lobe, PPC = posterior parietal cortex (Area 7 with body schema), VC = visual cortex, GnRH = gonadotropin-releasing hormone, FSH = follicle stimulating hormone, LH = luteinizing hormone, ICA = intercostals artery, T/L = thoracolumbar.

Right thoracic, but not left thoracic AIS in girls, is considered by Sevastik and colleagues to be initiated by dysfunction of the sympathetic nervous system leading through vascular changes to relative overgrowth of concave periapical rib lengths [59-63]. This section is written in collaboration with Professor JA Sevastik. Compared with right thoracic AIS, the pathogenesis of left thoracic AIS in girls remains relatively unexplored [[114,115], see DISCUSSION (6)]. The thoracospinal concept of pathogenesis was established from anatomical and clinical evidence including left-right asymmetries of thoracic skin temperature, breast size and vascularity, and periapical rib length asymmetry [30]. Subsequent experimental studies [61] provided evidence for the correction of experimentally-induced scoliosis consistent with the pathogenetic conclusions. The thoracospinal concept is supported by recent studies on breast size [183], vascular [184,185] and peripheral nerve [186] findings. It does not encompass evidence relating to the new neuroskeletal biology, energy homeostasis, or white adipose tissue which is central to the regulation of energy balance by adipokines, particularly leptin, hormones of the digestive system and metabolites, particularly glucose (Figure 8).

Biomechanical mechanisms are thought to be involved in pathogenesis. Evidence [60] showed that gradual elongation of one rib affects the position of the numerically corresponding vertebra in the three cardinal planes in a way similar to the apical vertebra in idiopathic scoliosis. The disc space wedging is explained by the rotational movement of the central vertebra in the frontal plane, and the lordotic tendency of the scoliotic segment is explained by ventral vertebral translation in combination with tilt in the sagittal plane. Curve progression is attributed to biomechanical mechanisms [63,80-82].

### New neuroskeletal biology (Figure 8)

In the last decade it was shown initially in mice, that the central nervous system regulates bone remodeling, and more recently longitudinal bone growth via the sympathetic nervous system linking leptin-responsive hypothalamic neurons to bone tissue [187-198]. In reviewing this new field of neuroskeletal biology, Patel and Elefteriou [195] summarize long-standing clinical observations relating to bone and the nervous system including reflex sympathetic dystrophy, hyperplastic callus associated with head injury and myelomeningocoele, and osteopenia associated with stroke, spinal cord injury and peripheral neuropathy. Conflicting reports on the effect of β-blockers for risk of fractures are published, and randomized clinical trials are needed [199]. Theoretically, neuroskeletal mechanisms expressed via the sympathetic nervous system *through its bilaterality *(Figure 5), could create asymmetries, although from animal experiments there is no evidence for or against such asymmetries.

### Energy homeostasis and sympathetic nervous system (Figure 8)

Bodily energy reserves are managed actively by complex systems that regulate food intake, substrate partitioning and energy expenditure thereby regulating long-term adiposity [200]. Energy homeostasis, fat and glucose metabolism are regulated by integratory centers in the central nervous system which receive, and convey information by signals from peripheral organs (such as adipocytes, gut and pancreatic islets - eg insulin and amylin both short-term satiety signals, the latter being a hind brain signal), and which send efferent neural and hormonal signals to peripheral tissues that regulate food intake, energy expenditure, metabolism and behavior (feeding) [200-203]. The obesity genes *MC4R*, *FTO and SH2B1 *may participate in the central control of energy homeostasis [172-174,200,203]. A neuroanatomical framework explaining the effects of leptin on neuroendocrine and sympathetic nervous system function has been reported [204].

### White adipose tissue, leptin, hypothalamus, sympathetic nervous system and bone formation/resorption in health (Figure 8)

Adipose tissue, where fatty acids are stored as triglycerides in lipid droplets, is central to the regulation of energy balance [205]. White adipose tissue constitutes separate depots that contribute with the hypothalamus as the key centre for integration and control of energy balance [200]. Leptin, best known as a satiety hormone, a signal of energy sufficiency and long-term adiposity, is one of several cytokine-like hormones secreted by adipocytes [1,2,200]. In girls there are gradual age- and BMI-related increases in circulating leptin levels [206]. Molna-Carballo et al [12] from a longitudinal study reported that the leptin concentration increases in both sexes with the progression of puberty, this value being 40% greater in girls, which correlates with the increase in body volume and fat accumulation [206,207]. Girls have higher serum leptin levels before, during, and after puberty than boys, even after accounting for the development of greater female adiposity [207]. The sexual dimorphism in leptin concentrations during puberty appears to be partly due to a stimulatory effect of estradiol on fat deposition and leptin concentration in females and a suppressive effect of testosterone on leptin concentration in males [207]. Leptin levels in men are lower than women at all decades of life [208].

Leptin, the product of the obesity gene (*ob*) circulates in both free and bound form, and targets neurons including the arcuate nucleus and other nuclei of the hypothalamus [200]. Leptin is a master hormone that acts via a specific receptor (OB-R with six types of receptor, LepRa-LepRf; the longest form, LepRb is the only receptor isoform that contains active intracellular signaling domains). The leptin receptor is present in a number of hypothalamic nuclei, where it exerts its effects. within a complex web of signals with many regulatory functions for food intake, body weight, increasing energy expenditure through sympathoactivation, thermogenesis, other metabolic and endocrine functions, reproduction, immune/inflammatory responses, and wound healing, mainly through signaling to the hypothalamus including [1,2,200,209]:

a) appetite repression and body weight control (*anti-obesity*, *anorexigenic);*

b) initiation of puberty in girls as one gate with *kisspeptin *in a permissive role [1,2]; genetic variation in LIN28B on chromosome 6 is associated with the timing of puberty [210];

c) stimulation of the sympathetic nervous system, more in females than in males, possibly because of their greater fat mass [211,212];

d) in bone formation, *anti-osteogenic in mice *acting centrally through the sympathetic nervous system [187-192,194-197,213] involving the molecular clock and circadian regulation [214], possibly with an opposite direct effect on bone [190,195,196,198]. Several genes are identified having high levels of expression in the hypothalamus [192,195,196]. Mice lacking β-adrenergic receptors have increased bone mass [215]. In feedback, the skeleton exerts an endocrine regulation of energy metabolism through the *Esp *gene exclusive to osteoblasts controlling secretion of the hormone-like substance osteocalcin [216-218] (Figure 8).

Animal experimentation suggests a two-way interaction between leptin and the sympathetic nervous system, with leptin causing sympathoactivation, and the sympathetic nervous system exercising regulatory feedback inhibition over leptin release [219].

### Leptin and bone growth in mice (Figures 8 and 9)

**Figure 9:**
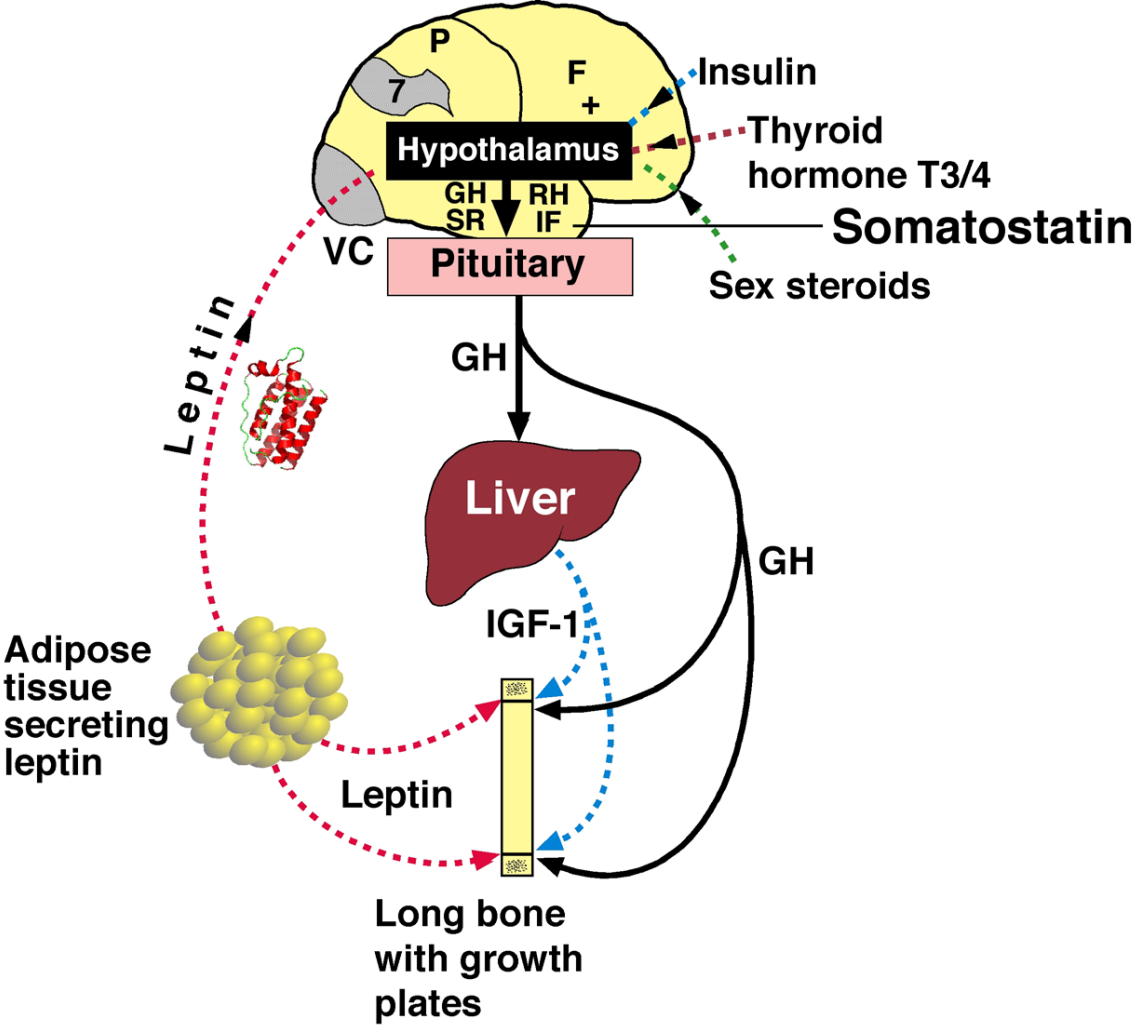
**Diagram showing three hormonal ways in which leptin stimulates growth plates: (1) GH by stimulating GHRH-producing neurons and inhibiting somatostatin-producing neurons, (2) IGF-1; and (3) directly**. [GH = growth hormone, GHRH = growth hormone releasing factor, SRIF = somatotropin release inhibiting factor (somatostatin) (Diagram modified from Gat-Yablonski and Phillip [222]).

Leptin stimulates longitudinal bone growth in leptin-deficient (*ob/ob*) and leptin-receptor deficient (*db/db*) mice [180,194,220-222], and growth plates in culture [180,222-224] being *chondro-osteogenic and angio-genic *[190]. The leptin appears to act centrally through the sympathetic nervous system (Figure 8) [190,194,213], growth hormone stimulation [180,190,220,222], and peripherally [190,222] with a direct effect on growth plate chondrocytes by its signaling receptor [180,220,222], regulating IGF-I receptor expression [190,223], and by other mechanisms (Figure 9) [180]. There is evidence for mice, that vertebral body growth plates may respond to leptin differently from long bone growth plates [179,180]. Iwaniec et al [194] propose that hypothalamic leptin plays a role in coupling energy homeostasis and bone growth, acting as an important permissive factor for normal bone growth. Leptin appeared in evolution with the bony skeleton [216].

### Leptin and bone growth in children

Maor et al [223] reviewed clinical evidence that after craniopharyngioma surgery in children, circulating leptin may contribute to bone growth including normal height velocity [225]. Children with exogenous obesity usually show increased height velocity [226], and their serum leptin levels are approximately five times that of normal children [227], with obese children being taller than average from 6-9 years [225], showing more advanced bone age/chronological age [227], earlier puberty and menarche [226] and no significant correlation of leptin and estradiol levels [228].

Montague et al [229] reported two severely obese consanguinous children with congenital leptin deficiency, the findings of which strongly suggested that leptin critically influences energy balance in prepubertal humans. One child developed abnormalities of growth in long bones of her legs treated by corrective surgery, an abnormality attributed to growth plate fragility [180]. Subsequently, in three children who were congenitally deficient in leptin and morbidly obese, Farooqi et al [230] reported radiological skeletal maturation was increased by 2.1 years, and that leptin therapy produced beneficial effects on the skeleton.

Severe dietary restriction, a common cause of leptin insufficiency and growth/length restriction in humans [194], is probably associated with, and explained by, decreased GH and IGF-I receptors in growth plates [231].

### Leptin, hypothalamus and AIS

Qiu and colleagues [163,164] reported a marked decrease in circulating leptin in AIS girls compared with controls, confirmed by Dr A Moreau (personal communication). Positive correlations were found between leptin and each of age, menstrual status, weight, corrected height, BMI, Risser sign, bone mineral content and bone mineral density (lumbar spine and femoral neck) but not Cobb angle, suggesting that leptin may play an important role in the lower BMI of AIS girls [164]. Longitudinal studies are needed.

### Central leptin resistance in obesity and possibly in healthy females

Central *leptin resistance *is defined as reduced ability of circulating leptin to suppress appetite and weight gain and to promote energy expenditure [232].

*In obesity*. Central leptin resistance isconsidered to be one of the main causes of obesity [232,233]. It is thought to result mostly from a state of diminished hypothalamic responsiveness to increased levels of circulating leptin [200] which may be selective [232-236].

*In healthy females: normal juvenile girls and somatotropic axis*. Central leptin resistance may occur normally in girls [227], and in pregnancy thereby permitting the accumulation of adipose tissue stores necessary for growth, reproduction and lactation [227,237]; leptin sensitivity returns, possibly by signaling mechanisms [232], or by altering the leptin dose-response curves [223,238]. There is preliminary evidence [50] suggesting that the hypothalamus of some normal juvenile girls, but not boys, functions with *central leptin resistance of the somatotropic (growth hormone/IGF) axis*. This putative mechanism, is interpreted as limiting energy invested in female skeletal growth thereby conserving energy for reproductive development [50]. It may be related to the female predisposition to AIS.

### Hypothalamic mechanisms of central leptin resistance in obesity

Several mechanisms have been revealed to explain central leptin resistance in obesity [232], namely:

(1) Impaired leptin transport across the blood-brain barrier e.g. *triglycerides *[238-240].

(2) Serum leptin interacting proteins (SLIPS) such as *C-reactive protein *[[241], but see [200]].

(3) Inflammation [239,242].

(4) Intracelluar inhibitory molecules (negative regulators) of leptin signaling including -

a) the *suppressors of the cytokine signaling *(SOCS) family [200,243,244],

b) *protein-tyrosine phosphatases *(PTPs) [200,245,246]; and

c) *OB-R gene related protein *(OB-RGRP) [247,248].

a) *Suppressors of the cytokine signaling *(SOCS). Howard et al [243] and Mori et al [244] noted that the leptin receptor is highly expressed in the hypothalamus and belongs to the cytokine-receptor superfamily that activates the Janus tyrosine kinase-signal transducers and the activators of transcription (JAK/STAT) pathway to modulate cellular responses in a negative feedback loop [[249,250], for detail and other pathways see [232]]. They report evidence for mice that *SOCS-3 *neuronal deletion enhances leptin sensitivity [244,250] as does haploinsuffiency of *SOCS-3 *[243]. *SOCS-3 *is also a human gene. *SOCS-2*, a genetic determinant of height growth in normal children, is involved in the regulation of IGF-I signaling [251].

*b) Protein-tyrosine phosphatases *(PTPs). PTP-1B also contributes to leptin resistance by inhibiting intracellular leptin receptor signaling by inhibiting JAK2 activation [232,240,252]. *PTP-1B deficient mice *by knockout and by an *antisense (anti-DNA) oligonucleotide *designed to blunt the expression of *PTP-1B*, showed improved leptin and insulin action [252]. *PTP-1B *is a major regulator of energy balance, insulin sensitivity, and body fat stores [246]. *PTP-1B *is also a human gene.

*c) OB-R gene related protein *(OB-RGRP). Couturier and colleagues [247,248,253] report that *OB-RGRP *negatively regulates the specific leptin receptor OB-R in the hypothalamus of mice. They comment that if the results obtained in the diet-induced obesity mouse model are transposable to humans, targeting the regulator of the leptin receptor rather than the receptor itself (either by RNA interference or by pharmacological antagonists), could be a more appropriate basis for identifying potential new therapeutic targets for a variety of diseases, including obesity.

(5) Intracelluar stimulatory molecules (positive regulators) of leptin signaling. According to Morris and Rui [232], SH2B1 enhances leptin signaling. It appears to be required for the maintenance of leptin sensitivity, energy balance and body weight, ultimately through activation of the PI 3 kinase pathway. The ability of SH2B1 to enhance leptin sensitivity may be modulated by other members of the SH2B family. Cellular leptin sensitivity may be determined, at least in part, by a balance between positive (e.g. SH2B1) and negative (e.g. SOCS3 and PTP-1B) regulators.

(6) Chronic endoplasmic reticulum (ER) stress, mediated through *protein tyrosine phosphatase 1B *and not through *suppressors of cytokine signaling-3 *[233], contributes to leptin resistance and obesity, presumably by activating various unfolding protein response signaling pathways, [232]. Inhibition of ER stress in the hypothalamus by either genetic or pharmacological means markedly improves leptin sensitivity and decreases food intake and body weight in mice [232].

(7) Defects in neural circuitry including impairment of MC4R signaling in the paraventricular nucleus, induce leptin resistance, hyperphagia and obesity, with genetic and environmental factors modulating the synaptic remodeling and rewiring of this circuitry [232].

The challenge is to develop diagnostic approaches for the different forms of central leptin resistance and design personalized healthcare programs to treat obesity [232].

### AIS as a systemic disorder - platelet calmodulin dysfunction [21,22,107]

Lowe et al [21,22] suggested that altered paraspinal muscle activity explained the relationship between platelet calmodulin level changes and Cobb angle changes in AIS with calmodulin acting as a systemic mediator of tissues having a contractile system (actin and myosin). An alternative speculative concept to explain the findings of Lowe is that in predisposed subjects, platelet activation with calmodulin changes occurs within dilated vessels of deforming vertebral bodies [107]. The activated platelets in juxta-physeal vessels release growth factors which, after extravasation, abet the hormone-driven growth of the already mechanically-compromised vertebral endplate physes to promote the relative anterior spinal overgrowth (*RASO*) and curve progression of AIS.

### AIS as a systemic disorder - melatonin, melatonin-signaling, osteopontin and soluble CD44 receptor

#### Melatonin deficiency

Machida and colleagues [7] found *lower plasma melatonin (MLT) levels *through 24 hours with progressive AIS curves and concluded that MLT disturbance has a role in AIS progression more than its cause. They suggested that AIS is an inherited disorder of neurotransmitters from neuro-hormonal origin affecting MLT associated with a localized neuromuscular imbalance and torsion in the bipedal condition [8,9]. The relevance of lower circulating MLT levels to AIS pathogenesis is now controversial since no significant decrease in circulating MLT levels has been observed in a majority of studies [254-256].

• MLT and leptin are said not to interact in the initiation or progression of human pubertal development [11].

• The relationship between MLT and GH is poorly understood [10,257].

• How MLT may interact with estrogens is discussed by Leboeuf et al [258].

• Melatonin-calmodulin interaction may represent a major mechanism for regulation and synchronization of cell physiology [22,259].

#### Systemic melatonin-signaling dysfunction

In progressive AIS, Moreau et al [14] found melatonin-signaling transduction to be impaired in osteoblasts, myoblasts and lymphocytes caused by the inactivation of Gi proteins. These findings, extended in subsequent papers [15-18], led to the conclusion that melatonin-signaling dysfunction detected in osteoblasts, myoblasts and lymphocytes is a decisive factor for the pathogenesis of AIS [17].

#### Osteopontin and soluble CD44 receptor

Most recently, Moreau et al [19,20] reported mean plasma osteopontin (OPN) levels to be increased in:

• patients with idiopathic scoliosis, correlating significantly with curve severity, and

• "an asymptomatic at-risk group" (offspring born from at least one scoliotic parent).

In contrast, mean plasma levels of soluble CD44 receptor (sCD44) were significantly lower in patients with Cobb angles of 45 degrees or more. Drawing on evidence from mouse models, it was concluded that OPN is essential to induce scoliosis formation and curve progression through interactions with CD44 receptors, *"thus offering a first molecular concept to explain the pathomechanism leading to the asymmetrical growth of the spine in idiopathic scoliosis." *[19].

We ask whether:

(1) in mice, the scoliosis of melatonin-deficient models has another interpretation; and

(2) in the AIS subjects [19,20], the increased OPN levels are secondary to bone remodeling.

### Some melatonin-deficient mouse models of scoliosis - markers of developmental stress?

Moreau et al [19,20] found all transgenic melatonin-deficient C57Bl/6J mice [150] devoid of OPN or CD44 receptor were protected against scoliosis, contrasting with wild-type ones. May this be, not because OPN is essential for scoliosis pathogenesis, but because OPN deficiency reduces stress reactions in mice [260]?

For, in mice, circulating OPN plays a significant role in the body's reaction to stress by regulating hormones of the *hypothalamic-pituitary-adrenal axis *(HPA) [260] modulated by leptin which activates the JAK/STAT pathway. Stressors cause less up-regulation of the stress hormone corticosterone in OPN-deficient mice [260]. This may be tested in the model used for mice: (1) rendered bipedal at 3 weeks of age, and (2) kept in tall cages to make them reach up increasingly for food and water [150]. The *developmental stress hypothesis *[261], if confirmed, suggests that OPN deficiency through reduced corticosterone up-regulation causes less stress-reaction damage to the neural development of posture and so protects against the scoliosis. If so, these transgenic mice findings [19,20] may not be relevant to AIS pathogenesis.

### Osteopontin and bone remodeling in mice

Osteopontin, a major non-collagenous bone matrix glycoprotein originally isolated from bone - sialic acid rich, phosphorylated and inhibitor of calcification - has a critical role in bone remodeling which in OPN-knockout mice was suppressed [262]. Hence, the interpretation under item 11. *above*, and the evidence from Fujihara et al [262], together raise caution about attributing a causal, rather than a consequential, role to increased plasma OPN in AIS pathogenesis.

### Melatonin receptor 1B (MT1B), AIS, glucose metabolism and type 2 diabetes

Promoter polymorphisms of the gene for melatonin receptor 1B (*MT1B*) are associated with the occurrence of AIS, but not directly with curve severity; this supports the hypothesis of a MLT-signaling pathway dysfunction in AIS [263]. There is a lack of association between promoter polymorphism of the *MTNR1A *gene and AIS [264]. Genome-wide association studies have shown that melatonin receptor 1B variation is also associated with insulin and glucose concentrations; the risk genotype of this SNP predicts future type 2 diabetes suggesting that blocking the melatonin ligand-receptor system in the endocrine pancreas could be a therapeutic avenue for type 2 diabetes [265,266]. These genetic findings:

• are consistent with hormone receptors having a variety of parallel but independent downstream effects; and

• raise the question: Do post-operative AIS girls after 60 years of age have a lower prevalence of type 2 diabetes, because they are protected by being leaner and using their energy in a different way with a more efficient burn within their systemic disorder?

## AUTONOMIC NERVOUS SYSTEM - leptin-hypothalamic-sympathetic nervous system (*LHS*)-driven mechanism in health and *LHS *concept in AIS (Figures 1, 4 and 5)

### Trunk widening in normal adolescent girls and the putative LHS-driven mechanism

We postulate that in normal girls, trunk widening of the pelvis, ribcage and shoulder girdle, characteristic of humans, is contributed to by a *leptin-hypothalamic-sympathetic nervous system (LHS)-driven mechanism *acting bilaterally (Figure 5). Differential sympathetic innervation between axial and appendicular bones may be present [196]. The pattern of skeletal sizes for age [47-49] suggests that any differential innervation by the sympathetic nervous system may differ between girls and boys.

**Figure 10:**
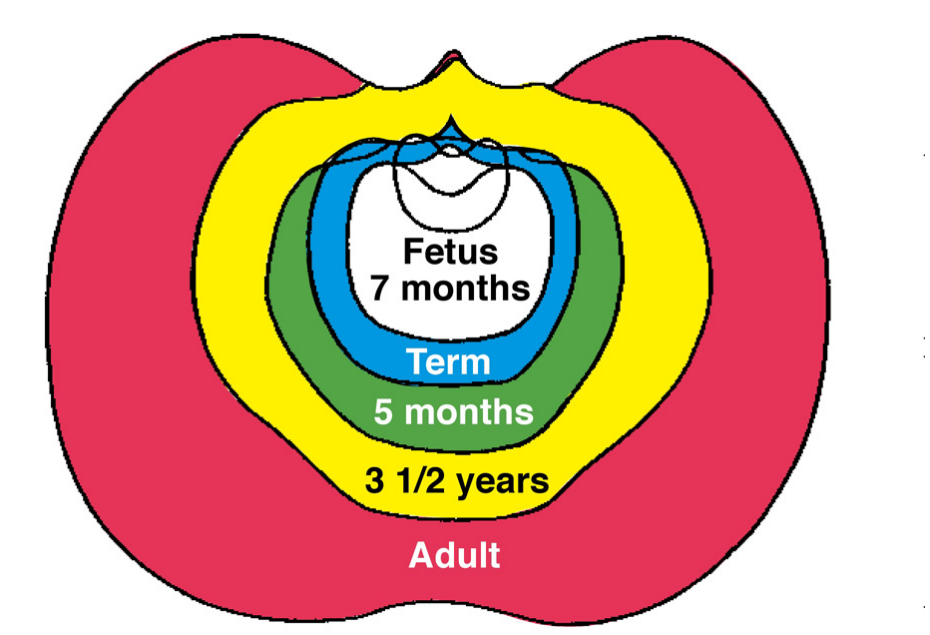
**Diagram of transverse sections of normal human thorax to show growth by age ranges: blue, fetus 7 months to term; green; term to 5 months; yellow, 5 months to 3 1/2 years; red, 3 1/2 years to adult**. Thorax width relative to depth increases mostly after 3 1/2 years (Modified from [268]).

**Figure 11:**
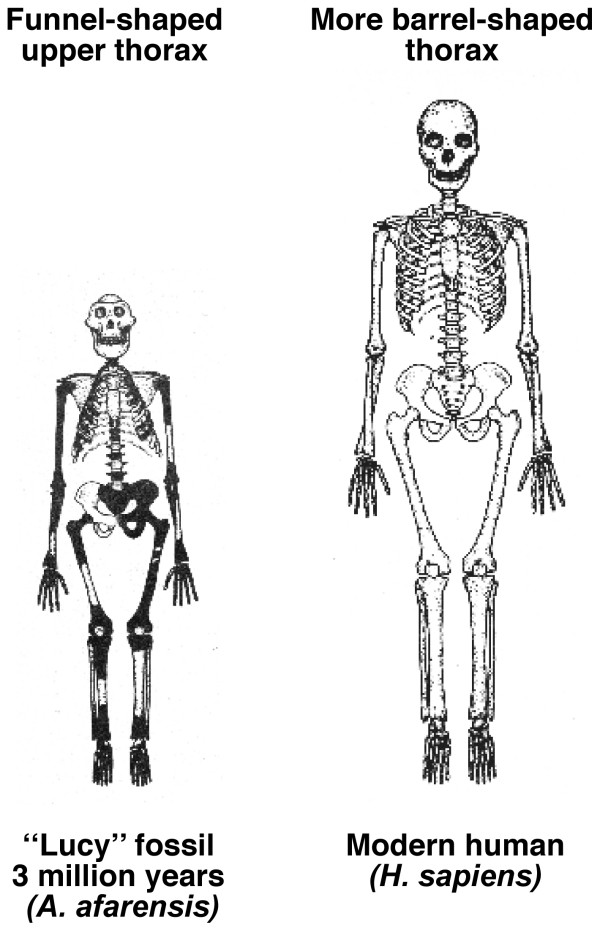
**The change in the ribcage from funnel-shaped to barrel-shaped in 3 million years of evolution**. Reassembly of the fossil skeleton (black) of *"Lucy" (Australopithecus afarensis) *compared with the skeleton of a modern human female. The upper thorax is funnel-shaped with narrow shoulders, like modern-day chimpanzees (Figure 12). The blades of the ilia have turned in providing hip mechanics appropriate for erect walking. Compared with the modern adult human female, "Lucy" was much smaller with the relative brain size of a chimpanzee, chimpanzee-shaped thorax, a broad pelvis from iliac flaring and widening of sacral alae (possibly related to gut size), and totally bipedal (Diagram modified from [269] and Burwell et al [149]).

**Figure 12:**
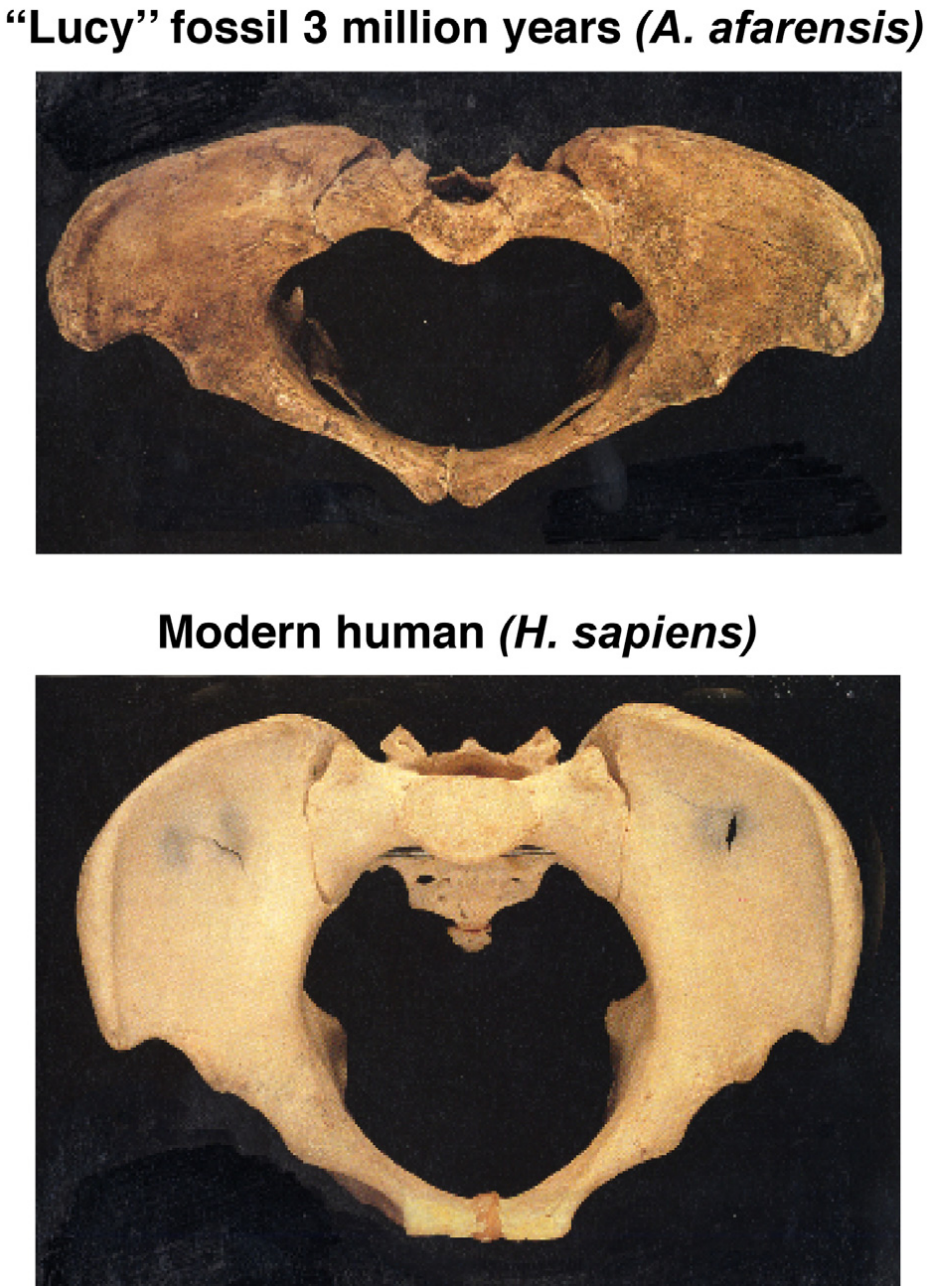
**Pelvis of "Lucy" and modern human female separated by 3 million years of evolution**. "Lucy's" sacral alae are wide thereby increasing separation at the hips, the ilia are more flared increasing the mechanical advantage for hip function, and frontal pelvic width greater than sagittal pelvic dimension. The major change visible in this view, namely the more ovoid form of the human pelvis, is accompanied by a sagittal expansion of the birth canal needed for the increase in brain size since "Lucy". (Modified from [271] and Burwell et al [149].

**Figure 13:**
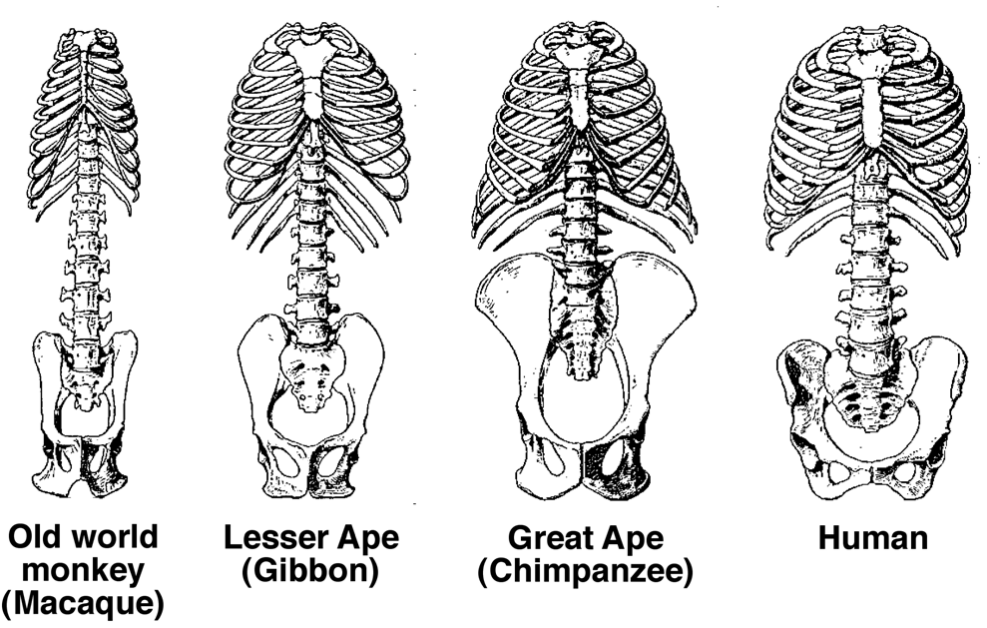
**Trunk skeletons of female primates reduced to the same total length**. Widening of the trunk - chest, shoulder and pelvis, is characteristic of all higher primates. Chimpanzees have an inverted funnel-shaped upper thorax with narrow shoulders. The human pelvis has increased in width mainly through great enlargement of its sacral portions but it is short as in monkeys [267] (Diagram modified from Schultz [267] and Burwell et al [149]).

**Figure 14:**
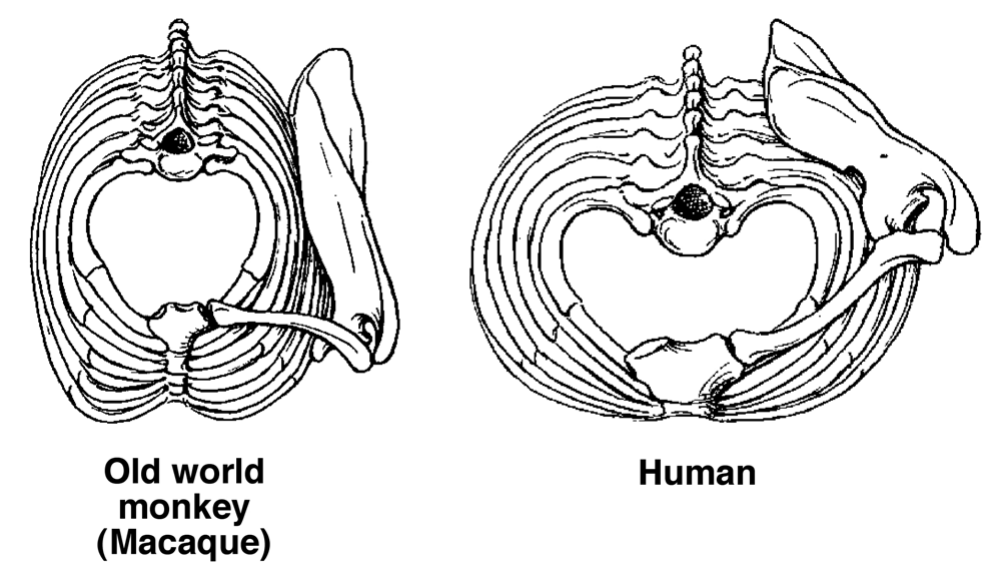
**Top views of thorax and left shoulder girdle in adult macaque and human**. In the macaque, the ribcage is narrow laterally and deep sagittally, while in truncally-erect forms it is expanded laterally and shallow from front to back, to keep the center of gravity over the feet. This trunk widening shifts the scapulae from the side to the back of the ribcage with clavicular lengthening, and the shoulder joints facing laterally rather than forward (Diagram modified from Schultz [267]).

In normal human growth, biacromial broadening reflects widening mainly of the underlying upper thorax (Figures 10 and 11) [149,267-269], and pelvic broadening reflects iliac flaring and widening mainly of the sacral alae (Figure 12); the latter reaches its maximum in hominins to provide a firm base of support for the trunk during bipedal posture and locomotion (Figures 13, 14) [153,267,269-271]. Hominid lumbar vertebrae also exhibit a caudally progressive widening of their laminae and of the space separating their articular processes [270]. Pelvic inlet width is a predictor of pediatric chest width [272].

The evidence suggests that pelvic widening in the frontal plane [267] (which varies with climatic conditions), together with pelvic incidence in the sagittal plane [273,274], provided hominins with conservation of energy [273] through biomechanical economy enabling -

• bipedalism with upright posture [75,153],

• modified spinal movements [275], and in the last 3 million years -

• increasing fetal brain size [270,271,276,277] with sagittal expansion of birth canal (Figure 12) [149,270,271], possibly with the bigger brain, from (1) a bigger baby,. (2) longer lumbar region, and (3) ability to conceive of tool construction and usage [276].

The evidence suggests that the medio-lateral dimension of the birth canal has been relatively (but not absolutely) ample since the australopithecine stage about 3 million years ago (mya = megaannum) with a funnel-shaped upper thorax (Figure 11) [269], as in the contemporary chimpanzee (Figure 13). A more ovoid pelvic shape with increase particularly of the sagittal dimension, then evolved in response to increasing brain size particularly from about 0.5 mya (Figure 12) [270,271] (see Evolutionary Origins).

### The LHS concept for girls with AIS

AIS in girls from the standpoint of the autonomic nervous system is viewed as expressing increased central leptin *sensitivity *of hypothalamic sympathetic functions and, in some girls, of the somatotropic axis, which subsequently develop an inverse relationship. We speculate that AIS arises from dysfunction of the normal *LHS-driven *mechanism (Figure 5) by genetically-determined and selectively increased hypothalamic sensitivity (*up-regulation from mutations*) to circulating leptin leading to hypothalamic asymmetry. The asymmetry is viewed as an adverse response to stress [25,36], with asymmetric activity mediated via the sympathetic nervous system bilaterally to vertebrae and/or ribs (Figures 1 and 5), to upper arm lengths in thoracic AIS, and to iliac heights in thoracolumbar and lumbar AIS. The increased sensitivity of the hypothalamus to leptin is viewed as being enhanced by increasing circulating levels of leptin from the fat accumulation of adolescent girls [12], despite the lower leptin levels of AIS girls [163,164].

The requirements for the theory are that in dysfunction, the sympathetic nervous system (SNS)-driven effects contribute with neuroendocrine mechanisms to produce [25]:

(1) Earlier skeletal maturation (hormonal).

(2) Sympathoactivation expressed asymmetrically in vertebral growth plates in 1-3 dimensions - left-right, front-back and/or torsionally - and in some paired bones (Figures 5 and 6).

(3) General skeletal overgrowth for age systemically distributed (hormonal)(Figure 7) [152].

(4) Left-right extra-spinal skeletal length asymmetries (ribs, upper arms and ilia) (Figure 1) with upper arm length asymmetry being a *signal *of thoracic vertebral and/or rib length asymmetry (Figure 6).

(5) Increased hypothalamic sensitivity to circulating leptin (*up-regulation*) involves the somatotropic (GH/IGF-I) axis [222] in some younger preoperative AIS girls (Figure 7, see Neuroendocrinology,. *Sympathetic nervous system and GH/IGF axis*).

(6) Hormonal effects of the GH/IGF axis cause exaggeration of the SNS-induced vertebral/rib length asymmetry contributing to curve progression of preoperative AIS girls in an inverse relationship (Figure 5, see Neuroendocrinology. *Sympathetic nervous system and GH/IGF axis*).

(7) Relative osteopenia [88,278,279] which results in part from sympathoactivation.

The lower BMI [163,164] and body fat of AIS girls may be determined genetically [172-174] and contributed to by sympathoactivation [176,219] from the putative hypothalamic *up-regulation *to leptin (*LHS concep*t) [25]. Overweight girls with AIS [170,171] probably reflect changes from genetic (leptin resistance in relation to satiety) and societal factors.

### Central leptin resistance/sensitivity and the LHS concept for AIS pathogenesis in girls

The LHS concept for AIS pathogenesis of girls, views the increased hypothalamic sensitivity to leptin as being at the opposite end of the spectrum to the central leptin resistance of obesity. This increased sensitivity to circulating leptin affects the *hypothalamic sympathetic nervous system *and, in some AIS girls, the *somatotropic neuroendocrine axis*. The effects produced in growing bones by these neural and endocrine mechanisms are influenced by the availability of energy, allocated by the hypothalamus through hormones and the nervous system, modulated by circulating leptin levels that measure long-term adiposity.

## Autonomic Nervous System - Possible Factors Causing Selective Hypothalamic Up-Regulation in AIS

We suggest five molecular mechanisms that might contribute to the selective up-regulation of some hypothalamic neurons to leptin in the *LHS *concept for AIS pathogenesis.

### G-protein coupled receptors

The putative dysfunction of hypothalamic neurons in AIS - increased and asymmetric sensitivity to leptin, may result from an abnormality of a G-protein-coupled receptor, or G protein, to leptin [25]. The melatonin-signaling dysfunction caused by the inactivation of Gi proteins so far detected is peripheral [14-20], and it is unknown whether any hypothalamic mechanism of etiopathogenesis is involved [Dr A Moreau personal communication]. Melanocortin-3 (MC3R) and MC4R are G-protein coupled receptors highly expressed in the hypothalamus [232].

### Circulating osteopontin (OPN)

Subject to the caveat expressed for circulating OPN levels having a causal role in AIS, increased levels of circulating OPN [19,20] may act as a gate for AIS in the hypothalamus as does *kisspeptin *for puberty through its G-protein-coupled membrane receptor GPR54 [2,280,281].

### Inhibitory molecules in the JAK/STAT pathway

Subject to the demonstration of a significant functional variation in human populations, *inhibitory molecules such as *SOCS-3 [232,243,244,250], PTB-1B [232,240,252]*and possibly the regulator of the leptin receptor *(OB-RGRP) [247,248,253] - all as negative regulators of leptin sensitivity, by their *decreasing *action, are candidates to *increase *hypothalamic sensitivity to leptin in the *LHS*-driven concept for AIS pathogenesis.

### Stimulatory molecules in the PI 3 kinase pathway

As positive regulators of leptin sensitivity, members of the SH2B family by their *increasing *action [232], are candidates to *increase *hypothalamic sensitivity to leptin in the *LHS*-driven concept for AIS pathogenesis.

### Hormesis - the putative cause of asymmetry in the LHS concept for AIS

*Hormesis *is a *bimodal dose response *to drugs and toxins, first stimulation and then an adverse response, usually inhibition [282-284]. There is evidence that this normal hormetic process applies to leptin [223]. The dose effect will be influenced by the combined effects of 1) increased hypothalamic sensitivity to leptin, and 2) raised circulating leptin levels from adolescent female fat accumulation. We speculate that in the hypothalamus the hormesis of leptin, in adversity leads *not to inhibition but to increased sensitivity and asymmetry *[36]. The concept is considered plausible by Dr EJ Calabrese [personal communication]. In rats, infused leptin increases sympathetic nervous system activity in a dose-dependent manner suggesting that leptin may act hormetically on the normal rat hypothalamus [285].

## Autonomic Nervous System - Rett and Prader-Willi Syndromes

### Rett syndrome

Rett syndrome is a genetic neuro-osseous developmental disorder much more prevalent in girls than boys, characterized by profound and progressive loss of intellectual functioning and growth failure [286,287]. Raised circulating leptin levels and overactivity of the sympathetic nervous system [288] are associated with its pathophysiology [286,287]. The skin sympathetic responses are related to the side of the scoliosis, on the foot ipsilateral to the convex side of the scoliosis where it shows a relatively lower amplitude [286]. These findings are consistent with the view that leptin and sympathetic nervous system dysfunction, under certain conditions, may be associated with scoliosis expression and curve laterality.

### Prader-Willi syndrome (PWS)

PWS, a rare multisystem genetic disorder, is thought to result from a central hypothalamic-pituitary dysfunction [289,290]. It is associated with failure to thrive in infancy and progressive hyperphagia and obesity in childhood; there is short stature with growth hormone (GH) deficiency, obesity, eating disorders, decreased muscle mass, hypotonia, hypogonadism, and a high prevalence of scoliosis in infants, juveniles and adolescents (15-86%) with 67% affected at skeletal maturity [289,291,292]. The pathogenesis of the scoliosis is unknown [293]; it is unrelated to gender and BMI [292] and may be related to decreased muscle mass, hypotonia, and hypo-excitability of motor cortical areas with defective neurogenesis of cortical tissue [294]. The contribution of the autonomic nervous system, if any, to the scoliosis appears to be unknown. PWS is not accompanied by deranged leptin concentrations and there was no evidence of an interaction of the GH/IGF axis with leptin metabolism in GH-deficient children [295]. While infants with PWS, have higher leptin levels than controls, suggesting a relative excess of fat to lean body mass [296], adults with PWS have leptin assessment corresponding to their degree of obesity [297] (see Endocrine and Therapeutic Implications, *GH treatment and the Prader-Willi syndrome (PWS)*).

## Evolutionary Origins

From the initial chimpanzee-human divergence about 5-7 mya, hominins may have evolved their loss of body hair by about 3.3 to 1.2 mya and its replacement with increased subcutaneous white adipose tissue (80% of all fat) for insulation and energy stores, more in maturing females than males [267,298-302]. About 2 mya, these changes were associated with the decoupling of head and trunk movements required for endurance running to hunt down prey [303], since when the hominid lineage leading to modern humans evolved significantly larger, and more sophisticated brains, than other primates [299-302].

### Melatonin decrease - the turning point of human evolution?

Explanations of "what makes us human" often include a bridge between culture and biology [51]. Recently, it has been suggested that decreased circulating melatonin levels due to light from campfires extending the day, "changed the timetable of growth, development and reproduction, because sitting by the fire altered the night's flow of melatonin and the cascade of hormones that follow it." [304].

### Fat - Brain Growth and Nutritional Stresses

Power and Schulkin [301] in their book, *'The Evolution of Obesity'*, outline an evolutionary hypothesis in relation to fat and hominin brain growth [299,300]. The book is one of the first to use an evolutionary framework to analyse a major body of neuroendocrine knowledge about a specific condition [53]. Power and Schulkin write:

*"Human beings have evolved to become very good at storing fat; fat appears to have been very important in our evolution. For example, human babies are among the fattest of all mammals... ...The importance of fat, both in our diet and on our bodies, appears to have increased in human beings compared to our nonhuman primate relatives. We suggest that this change in nutritional biology was linked to the seminal evolutionary event in our lineage: our larger brain." *[301].

Nutritionally, human brain growth is said not to be costly [299], but it does require docosahexaenoic acid (DXA), present in body fat more at birth than at any other time in life [300]. The functioning human brain enlarging particularly in the first two years of postnatal life, imposes a burden on metabolism by -

• increasing energy demands, and

• restricting flexibility in energy allocation when nutritional supply is disrupted - as in the nutritional stresses of weaning and childhood infections [299,301,302].

The relation of leptin to brain growth is not considered here [133].

### Fat - Trunk Width Growth and the LHS Normal Mechanism

We suggest that another *'seminal evolutionary event' *- earlier in our lineage than brain growth, was trunk width growth which has increased more in human beings compared with our nonhuman primate relatives; the latter lack the extended childhood and rapid and large acceleration of growth velocity at adolescence in humans (Figures 11, 12, 13, 14) [153,267,270,271,303].

• *Pelvic width*. In hominins, increased pelvic as iliac and sacral width for habitual erect walking was established by about 3 mya (Figure 12).

• *Thorax and shoulder gitrdle width*. Ribcage widening, particularly of the upper thorax (Figure 11) happened in the last 3 million years. The wide shoulders characteristic of *Homo *[303] evidently resulted from upper ribcage widening relative to depth (Figures 10 and 11), with clavicular lengthening (Figure 14). This trunk widening at the shoulder girdles is likely to have been selected by:

a) the evolution of upright posture giving an enhanced respiratory importance to the upper thorax [see [268]]; and

b) counter-rotations of upper thorax and arms (but not the head) providing counter-balancing torques generated by shoulder girdles and arm-swinging needed to oppose torques created by the pelvic rotations of hominin bipedalism [71,149,268,303].

• *Brain and pelvic depth*. The large *fetal *brain size enabling a dramatic jump of adult brain size from about 0.5 mya, was made possible by further expansion of the birth canal, particularly sagittally (pelvic depth) (Figure 12) [75,267,299-303].

The *LHS mechanism *suggests that the fatness of hominins, starting over 3 mya, raised circulating leptin levels which, through the hypothalamus and sympathetic nervous system, supplemented the hormonally-driven growth in width of pelvis and ribcage [26] (Figures 10, 11, 12), and not in nonhuman primates [153,267] (Figures 5, 13 and 14). This mechanism, we suggest, provided a process in evolution that contributed to:

• pelvic widening mainly from sacral widening, enabling bipedalism with upright posture, later

• upper thorax with shoulder widening, and still later

• increased pelvic depth of *Homo sapiens *(Figure 12).

The *LHS mechanism *is interpreted as being evident today in normal human development as *'energy priority of trunk width growth' *in girls (Figures 4 and 5) [47-49].

We speculate:

• In evolution, to reduce toxicity to the hypothalamus of the raised circulating leptin levels - signaling greater adipose tissue stores particularly in females, hypothalamic sensitivity to circulating leptin became *diminished *(*desensitized, or down-regulated, i.e. central leptin resistance*), possibly involving *increased *action of *inhibitory *molecules such *as *SOCS-3 and PTP-1B, or *decreased *action of *stimulatory *molecules such as SH2B1. It needs to be established whether humans deal with SOCS-3, PTP-1B, and SH2B1 differently from other apes.

• In evolution, the development of human bipedalism and upright posture necessitated adaptations of postural control by the somatic nervous system [51].

• *The putative central leptin resistance in the somatotropic (GH/IGF) axi*s of normal juvenile girls [[50], see [227,237]] is linked to a greater evolutionary *down-regulation *to leptin in the female than the male hominin hypothalamus.

### Fat - AIS in Girls and the LHS Concept of Pathogenesis

The *LHS concept for AIS *pathogenesis suggests that the putative genetically-determined selectively increased hypothalamic sensitivity (*up-regulation from mutations*) to leptin leading to hypothalamic sympathetic asymmetry is rooted in the evolutionary origins of hominin fat deposition providing the energy needed for trunk width growth and later, brain growth and metabolism. We posit that increasing levels of circulating leptin associated with fat accumulation of adolescent girls [12], enhance the putative increased hypothalamic sensitivity (sympathetic and somatotropic) to leptin of AIS girls. This raises the question: Is the societal fat accumulation of normal adolescent girls [156] associated with increasing severity [170,171] and/or prevalence of AIS?

Left-right asymmetries of the neuroendocine system and of hypothalamic structure and sex-linked function are reported in normal animals [305].

## Endocrine and Therapeutic Implications

Within the somatic nervous system the *escalator concept*, at present, does not provide any new therapy to improve postural control for early AIS. In contrast, in the autonomic nervous system, the *LHS concept *for AIS pathogenesis suggests two broad therapeutic strategies: through the hypothalamus, and neuroendocrinology.

### Hypothalamus

Badman and Flier [200] state that the *improvement *in central leptin signaling by PTP-1B may provide a target for pharmacological intervention for weight-loss therapies [306]. Similarly, the *LHS concept for AIS* pathogenesis suggests that *impairment *of central leptin signaling may ultimately provide a target for pharmacological intervention for progressive AIS in girls, if this can be done selectively.

### Neuroendocrinogy

#### Sympathetic nervous system and GH/IGF axis

The *LHS concept *suggests manipulatable causes for therapy (Figure 5) relate to:

(1) sympathetic nervous system causing asymmetries in spine, trunk, upper arms; and

(2) increased levels of circulating growth hormone (GH)[136,307,308] for age in AIS girls notably from 7-12 years, and in pubertal stage 2, and/or IGF-I formerly known as somatomedin C [5,309].

Item (2) may exaggerate the putative sympathetic nervous system-induced vertebral asymmetry particularly in prepubertal and early pubertal growth and thereby contribute to curve progression (Figure 5). Hormonal involvement in AIS progression is supported by the finding that the initiation of the *curve acceleration phase *correlates with the timing of peak height velocity and simultaneously with digital changes in bone aging (400-425 of the Tanner-Whitehouse RUS III method, stage F covered phalangeal epiphysis to G capped phalangeal epiphysis [5]).

The GH/IGF axis is the pivotal system [310] with estrogen [311] for regulating axial growth during puberty. Evidence from normal juvenile girls with relatively higher BMIs suggests there is *central leptin resistance in the somatotropic axi*s [[50], see [227,237]] which, through mutations causing central leptin sensitivity, may predispose some girls to AIS. Several papers suggest that the GH/IGF axis has a role in the pathogenesis of AIS [310,312,313], with IGF-I polymorphism affecting curve severity of AIS but not its onset [314]. Growth hormone treatment may increase the risk of progression of scoliosis [315-318].

We suggest that in preoperative AIS girls with relatively higher BMIs, the skeletal overgrowth for age (Figure 7) [38,39,41,135-141,152] results from earlier and *increased hypothalamic sensitivity of the GH/IGF axis to leptin for age *leading to increased GH/IGF secretions, and possibly estrogen through other neuroendocrine axes. In the lower BMI subset of preoperative AIS girls, there is no early and systemic skeletal evidence to suggest increased secretion of GH/IGF-I (Figure 7) According to the *LHS concept*, more sympathoactivation in the lower BMI subset is needed to account for curve magnitudes which are similar to those of the higher BMI subset (Figure 7). This interpretation implies that in AIS girls, GH/IGF axis secretion and sympathoactivation may have an inverse pathogenetic relationship (Figure 5, see Discussion, *Medical conditions showing inverse relation of GH/IGF axis secretion and sympathoactivation*).

The therapeutic implication for AIS girls is that, whatever the BMI, consideration be given, early in curve evolution, to decreasing -

• growth hormone and IGF synthesis by a *somatostatin analogue *as used in tall children [319] (Figure 9), and/or

• sympathetic nervous system activity by β-blockers (as being evaluated for fractures [199]) (Figures 5 and 8).

Either medication, separately or together, might decrease vertebral and/or rib asymmetry and limit scoliosis curve progression, possibly by also affecting bone remodeling [199]. This strategy ignores a possible role for sex hormones in pathogenesis.

#### GH treatment and the Prader-Willi syndrome (PWS)

That GH may increase the risk of scoliosis progression is currently being evaluated in PWS patients having GH treatment for the short stature [290,292,320,321]. In the first study of a large population of children with PWS treated with GH, beneficial effects were found with no adverse effects on the progression of scoliosis [321]. In the light of the *LHS concept for AIS*, the latter finding suggests that in PWS, vertebral growth asymmetries are not primarily involved in the cause of its scoliosis, which may reside in musculature and somatic nervous system.

### Sex hormones

#### Estrogen and testosterone

A third potentially manipulatable cause of AIS pathogenesis in girls relates to sex hormones in pubertal growth [17,258,311,322,323]. The relation of age at menarche to peak height velocity in AIS girls [5,6,258] and genetic findings [324-326] suggest a role for estrogens in susceptibility and/or curve progression. In the *LHS concept*, estrogens like GH, may exaggerate vertebral growth plate asymmetry and curve severity particularly in girls with relatively lower BMIs (Figure 7). Circulating levels of estrogen are reported to be normal or lower, and of testosterone raised, in AIS girls [307,327-329].

#### Gonadorhelin analogues

**Figure 15:**
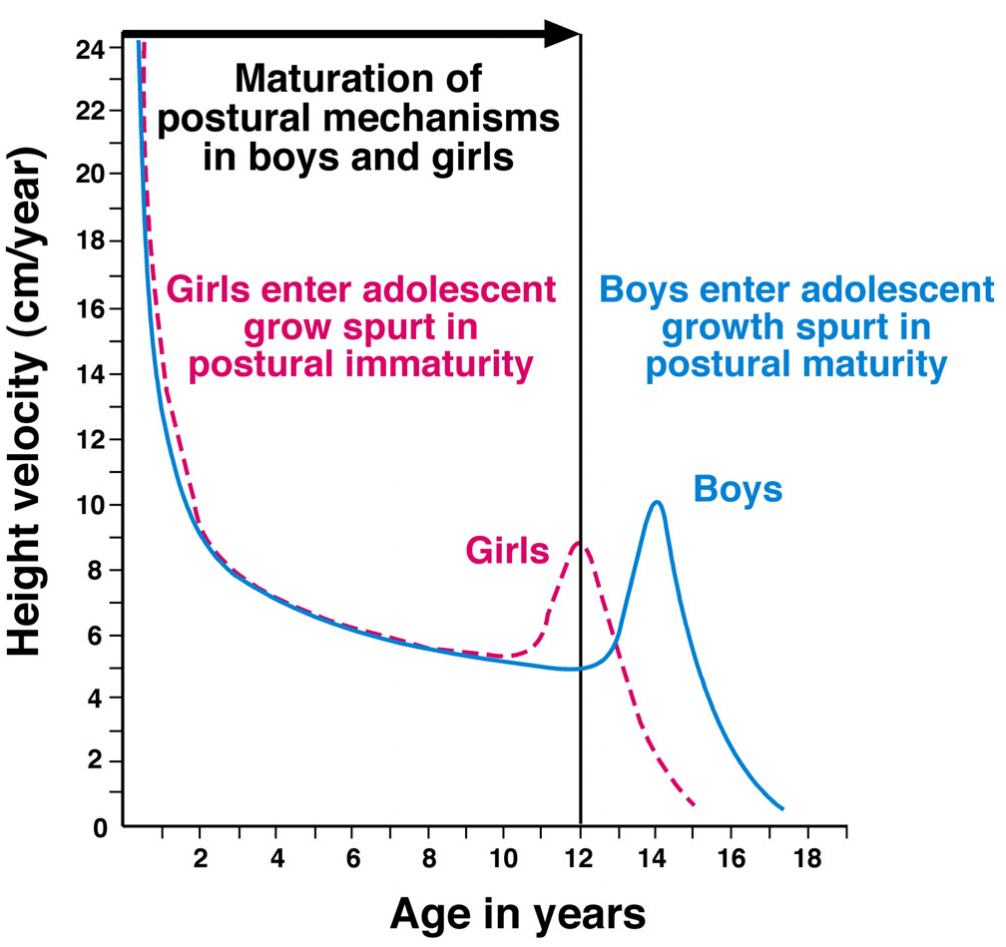
**Neuro-osseous timing of maturation (*NOTOM*) concept to explain the female susceptibility to progressive AIS in relation to the somatic nervous system**. Height velocity (cm/year) is plotted against age in relation to putative postural maturation at 12 years of age in both sexes. The postural immaturity of girls due to their earlier growth spurt makes them more susceptible to curve progression than boys. A curve initiating factor is not identified in this concept. The age and sex effect of postural sway in healthy children needs further evaluation [71]. (Diagram modified from Burwell and Dangerfield [330-332]).

The *NOTOM *concept (Figure 15) [71,330-332] suggests a medical treatment for AIS, by administering a *gonadorhelin analogue *(Figure 8) to delay menarche and slow bone growth in early AIS [333] - as practised for children with *idiopathic precocious puberty*. This is not an ideal option, as delaying the timing of normal puberty adversely affects bone mineralisation, and potentially could increase the risk of osteopenia long-term.

#### Ballet dancers, hypoestrogenism and leptin

The increased prevalence of mild right thoracic scoliosis in ballet dancers is associated with delayed menarche, secondary ameorrhea, anorectic behavior, osteopenia, fractures and prolonged hypoestrogenism [334]. The *LHS concept *for AIS pathogenesis applied to the scolioses of ballet dancers suggests that presumed low leptin levels [335] are associated with:

(1) increased selective hypothalamic sensitivity to leptin;

(2) increased sympathoactivation with asymmetry expressed in the spine as scoliosis;

(3) limited energy being diverted away from the *gonadotroph-gonadal axis*, possibly also the *hypothalamic-pituitary-adrenal axis *[335] and *GH/IGF (somatotropic) axis*; and

(4) osteopenia and fractures.

Treatment for the menarcheal delay includes oral contraceptive therapy [335].

### Melatonin-signaling dysfunction [12-17]

Other manipulatable causes of AIS pathogenesis are suggested by the *melatonin-signaling dysfunction *detected in osteoblasts and chondrocytes.

*(1) Osteoblasts*. *In vitro*, MLT significantly *stimulates osteoblast proliferation*, differentiation and mineralization from controls [336], but not in osteoblasts from AIS subjects [337,338]; this defect is suggested to play a role in the low bone mineral density of AIS patients and contribute to pathogenesis [338].

MLT-signaling dysfunction in AIS subjects has been revealed mainly using bone tissue because (a) osteoblasts respond to MLT, and (b) relative osteopenia is often observed in patients with AIS [14,15,88,278,279]. In some girls with AIS, a particular MLT-signaling defect is evident [17,256,258,337]. Correction of this defect *in vitro *by estradiol suggested that "the lack of estrogen that results in late menarche may be corrected by estrogen agonists having a positive effect on bone tissue remodeling" [256]. Leboeuf et al [258] suggest estrogens as important pharmacological targets to consider in AIS therapy directed to patients selected on their tissue response to MLT. This is in contradistinction to the suggestion of delaying the adolescent growth spurt for subjects in the lower BMI subset using a *gonadorhelin analogue *[330-333] (see Sex hormones).

*(2) Chondrocytes*. In cartilage from controls, MLT significantly *inhibits chondrocytes proliferation in vitro *but not from AIS subjects [339]. According to Wang and colleagues [339], the non-responsiveness (*i.e*. lack of inhibition) of AIS chondrocytes to MLT might play a role in the abnormally increased bone growth of AIS girls from dysfunction of the MLT-signaling pathway. In this connection, there is a decreasing expression of MT1 and MT2 mRNA in chondrocytes from AIS patients which may be related to the molecular pathogenesis of AIS [340].

### Research needs

Rather than a clinical trial of a *somatostatin analogue *and *β-blockers*, we suggest that currently there is a need to evaluate circulating hormones and sympathoactivation in AIS girls by relatively higher and lower BMI subsets.

In addition to using *cellular dielectric spectroscopy *for AIS diagnosis based on G-protein coupled receptor detection [18], Moreau et al [19,20] suggest OPN and sCD44 as useful markers for diagnosis and prognosis of idiopathic scoliosis. Subject to further study, as already mentioned, OPN may be a potential target for therapeutic intervention in AIS subjects as suggested for psoriatic patients [341] (see *Some melatonin-deficient mouse models of scoliosis - markers of developmental stress?*).

## Discussion

### Abnormalities revealed by higher and lower BMI subsets for AIS girls

The analysis of our skeletal data by relatively higher and lower BMI subsets distinguishes two types of effect: skeletal sizes for age (Figures 4 and 7), and skeletal asymmetries (Figure 6).

*Skeletal sizes for age - energy priority of trunk width in girls*. The skeletal size for age effect in the girls is shown as differences between -

*(1) higher and lower BMI subsets *in each of preoperative, screened and normal girls (Figures 4 and 5) restricted mainly to the trunk [46,117-119]; and

*(2) preoperative and normal girls *in higher and lower BMI subsets (Figure 7).

The trunk width growth priority of girls is seemingly a human characteristic. It is not explained by any of the prevailing theories of AIS pathogenesis (Appendix 1, items 1-15) each of which solely addresses pathogenesis. The trunk width features are accommodated by the *LHS mechanism *which invokes the sympathetic nervous system and hormones (Figure 5).

#### Skeletal sizes for age - curve severity, sympathoactivation and hormonal stimulation

In both higher and lower BMI subsets of preoperative AIS girls, mean Cobb angles are similar (Figures 4, and 7) with similar mean ages and curve types. It could then be argued that BMI is irrelevant to AIS pathogenesis. But the earlier systemic skeletal overgrowth for age of the *higher BMI subset *of younger preoperative girls (Figure 7), suggests that abnormally increased hormonal stimulation ?GH/IGF secretions, is associated with AIS pathogenesis. This led to the hypothesis that GH/IGF secretions exaggerate the sympathetic-induced vertebral and/or rib asymmetry and increase scoliosis severity.

The *lower BMI subset *lacks evidence of earlier systemic skeletal overgrowth for age (Figure 7). In this subset, we postulate that less GH/IGF axis secretions are associated with more sympathoactivation in an inverse relationship (Figure 5). The combined sympathetic-hormonally-induced effects in the lower BMI subset produce mean Cobb angle and mean upper arm length asymmetry similar to, and mean AVR less than, the higher BMI subset (Figure 6) [46]. This postulate of an inverse relationship ignores other possible mechanisms that may contribute to curve progression common to each BMI subset, including osteopenia [88,278,279], biomechanical spinal growth modulation [80-82], intervertebral disc degeneration [45,342-351], and platelet calmodulin dysfunction [21,22,107].

#### Medical conditions showing inverse relation of GH/IGF axis secretion and sympathoactivation

Several conditions in health and disorder show an inverse relationship of GH/1GF secretions and sympathoactivation. GH/IGF (somatotropic) axis secretions are associated with central sympathetic outflow [352,353] in an inverse relationship, though not for physical exercise [354]. In well-nourished subjects under basal conditions, evidence for an inverse relationship of GH secretion and sympathoactivation includes: acromegaly [355,356], GH-deficiency in adults [352,353,357], GH treatment of GH-deficient adults [353], idiopathic cardiomyopathy [358], middle-aged men with high waist-hip circumference ratios with reduced GH peak size concentrations [359], ageing men, with declining GH and IGF-I secretions [360], and growth hormone transgenic mice [356].

The need for this inverse relationship under basal conditions is shown by the following:

(1) In well-nourished subjects, GH stimulation of IGF and insulin is important for the anabolic storage and growth of adipose tissue, glycogen reserves and lean body mass [361]. In fasting, other catabolic states and stress, GH is lipolytic, liberating free fatty acids as an energy source.

(2) The sympathetic nervous system and catecholamines are key components of lipid mobilization in stress [362,363].

#### Skeletal asymmetries and lower BMI subsets

In the *lower BMI subsets *skeletal asymmetries are found in:

(1) preoperative girls upper arm length asymmetry is significantly greater than in screened and normal girls (each p < 0.001) [46]; and

(2) right thoracic AIS, wherein Cobb angle and apical vertebral rotation are each significantly associated with upper arm length asymmetry but only in the lower BMI subset (Figure 6) [46,120,121].

The abnormally increased upper arm length asymmetry with right thoracic AIS is explained by the *LHS concept *as resulting from the sympathetic-induced asymmetric effect on humeral linear growth. This asymmetry is not significantly different in magnitude between *lower and higher BMI subsets*. It is limited to proximal upper limbs (brachium), putatively to ribs and vertebrae, all putatively influenced by hormonal effects ?GH/IGF.

#### Upper arm length asymmetry and the higher BMI subset of right thoracic AIS

In the higher BMI subset of girls with right thoracic AIS, upper arm length asymmetry decreased significantly with age. The *LHS concept *explains this resolution as sympathetic- and hormonally-induced asynchronous upper arm growth affecting either:

(1) younger more than older adolescent girls; or

(2) all girls *transiently*, with the asymmetry starting in late juvenility with vertebral and/or rib length asymmetry that triggers the scoliosis.

Any associated vertebral osteopenia, possibly sympathetic- and/or hormonally-induced, may then predispose to curve progression. Any transience of the upper arm length asymmetry may result from the *neuroprotective action *[132] of rising circulating leptin levels during the early stages of puberty [206-208]. This could reduce the breadth of hypothalamic asymmetric dysfunction, which may not occur in the lower BMI subset with presumptively lower circulating levels of leptin producing less *neuroprotection *with a tendency to more asymmetry.

### Explanations for undisputed facts about AIS

Theories about the pathogenesis of AIS have to explain several undisputed facts [91,110,364].

(1) *Dependence of the deformity upon growth and growth rate*. The relation of skeletal growth velocity to curve progression in AIS is established [4,5,137,365,366], but its mechanism of action is unclear - causative, conditional, amplifying, or coincidental [91]. In the *escalator concept*, the dependence of AIS progression on growth is explained not by velocity of growth, but by rapid spinal lengthening and trunk enlargement beyond the capacity of the postural mechanisms to control the deformity [24,51,111].

(2) *Predilection for females*. Two putative mechanisms explain the greater susceptibility of girls than boys to progressive AIS:

a) In the autonomic nervous system, the *increased sensitivity *(*up-regulation) of the *hypothalamus (sympathetic NS and somatotropic axis) *to leptin by mutations *with its asymmetries contributing to AIS, greater in females than in males [25], is attributed to: i) *diminished sensitivity (down-regulation, i.e. resistance) to leptin *of the female hypothalamus established by mutations in hominin evolution; and ii) *central leptin resistance in the somatotropic axi*s of normal juvenile girls [50] which, through mutations causing central leptin sensitivity, may predispose some girls to AIS.

b) In the somatic nervous system, girls may enter their adolescent skeletal growth spurt in postural *immaturity*, compared with boys who may enter their adolescent growth spurt in postural *maturity *so they are protected from developing a scoliosis curve (Figure 15) [330-332].

(3) *Involvement of members in involved families*. This is determined by genetic factors operating in the autonomic and somatic nervous systems [56,77-79] and other mechanisms.

(4) *Curve types and laterality patterns*. Biomechanical factors involving ribs [59-63] and/or vertebrae [64,65,91-93] and spinal cord [64,65,92,93], acting during growth may localize AIS to the thoracic spine and cause the sagittal spinal shape alterations [83-90]. The non-random laterality of thoracic AIS curves has been explained by several factors including handedness, aorta, lungs, diaphragm, pre-existing lateral curve, axial rotation and embryology [367-371]. We suggest that the laterality and site of **t**horacic, thoracolumbar and lumbar curves is determined, in part, by the location of the putative abnormalities of the *LHS-driven *mechanism in the hypothalamus and sympathetic nervous system.

(5) *Varied progression patterns*. These are explained by the interaction of autonomic and somatic nervous systems in the spine and trunk compounded by any relative osteopenia of vertebrae [88,278,279], biomechanical spinal growth modulation [80-82], accelerated disc degeneration [45,342-351], and platelet calmodulin dysfunction [21,22,107]. Circulating leptin levels in AIS girls did not correlate significantly with Cobb angle [163,164]. This finding does not preclude circulating leptin levels acting with increased hypothalamic sensitivity to leptin to contribute to the magnitude of the hypothalamic asymmetry, and from that to the sympathetic nervous system-induced skeletal asymmetry(ies).

(6) *3-D rotatory deformity of the spine*. In thoracic AIS, Davids et al [372] found that the most valuable single MRI indicator for abnormal central nervous system findings was the absence of an apical segment lordosis. This and other evidence [91,373] suggests that in thoracic AIS, apical lordosis [83-87] is determined by processes either intrinsic to the spine ("primary", *i.e*. relative anterior spinal overgrowth = RASO [51,76]), and/or extrinsically by the sympathetic nervous system acting on vertebrae in 1-3D - left-right, front-back, and/or torsionally. Recent evidence shows that while right thoracic AIS has a reduced thoracic kyphosis (T5-12), increased pelvic incidence and sacral slope consistent with the RASO theory of pathogenesis [374], left thoracic AIS [374] has a normal thoracic kyphosis and pelvic incidence, not consistent with the RASO theory. This may signify that left thoracic AIS has a pathogenesis different from right thoracic AIS [374], possibly involving reduced white matter density of the central nervous system [114,115]. We suggest that right and left thoracic AIS in girls may be driven separately by the two nervous system components of the double neuro-osseous theory: right thoracic AIS mainly by the autonomic/sympathetic nervous system and left thoracic AIS, mainly by the somatic nervous system.

(7) *Vertebral bodies grow faster than the posterior vertebral elements *[64,65,83-90]. This is explained in part by a greater enhancing effect of the sympathetic nervous system on vertebral bodies and their growth plates than on posterior vertebral growth leading to asymmetry in the sagittal plane and the relative anterior spinal overgrowth (RASO) of progressive AIS.

(8) *AIS is exclusive to humans*. We suggest that AIS in girls is a consequence of abnormalities occurring in the putative physiological *LHS-driven *(*Figure *5) and *escalator (Figure *2*) mechanisms *of the theory, both of which are unique to humans [24,25,50] and emanating from these and other features of their evolution [298-303].

## Testing the Theory

The double neuro-osseous theory (Figure 1) cannot be tested as a singularity, but many of its components, framed as hypotheses, can be tested by refutation within ethical restraints. In the multidisciplinary approach needed, some problems to be addressed include the following.

(1) Genetic factors operating in somatic and autonomic nervous systems may be investigated in members of families with AIS girls, by genome-wide association studies in relation to postural control data [94] and objective evidence of autonomic dysfunction respectively (see below item (12)).

(2) Studies of brain imaging, function and asymmetries of AIS subjects compared with normals during adolescence need to be extended [113-115,375]. A basic question to be addressed is: Is the spinal and trunk deformity of AIS in girls the solitary expression in the spine and trunk of a brain that is the seat of several abnormalities of symmetry control?

(3) By relatively higher and lower BMI subsets, confirmation is needed for *energy priority of trunk width size *for age in normal and AIS girls (Figures 4 and 5), skeletal asymmetry growth patterns in girls with thoracic AIS (Figure 6), and skeletal overgrowth patterns for age in preoperative/normal girls (Figure 7). In normal babies, evaluate skull size and trunk width by relatively higher and lower BMI at each of birth, one and two years of age [376,377].

(4) By relatively higher and lower BMI subsets confirmation is needed of evidence suggesting *central leptin resistance in the somatotropic *(GH/IGF) *axis *of normal juvenile girls [50] which, through mutations causing central *leptin sensitivity*, may predispose some girls to AIS. The possibility of other mechanisms explaining the findings needs to be evaluated by studies of leptin, soluble leptin receptor and free leptin index [378,379].

(5) Since bilateral skeletal asymmetry in humans and skeletal overgrowth for age may be the key factors for the development of AIS [76], etiopathogenetic research needs to focus on skeletal length asymmetries of normal and AIS girls (Figure 1), and their relation to each of skeletal size for age, and osteopenia. The evolution of upper arm length asymmetry in girls with right thoracic AIS [135] and normal right thoracic trunk asymmetry [123-125] needs to be established in longitudinal studies of higher and lower BMI subsets.

(6) In leptin-deficient *ob/ob *mice, evaluate whether vertebral growth plates respond to absent leptin signals in a fundamentally different manner from limb bone growth plates [179,180].

(7) The energy sources of growth plates (GPs) in the trunk and limbs of humans and quadrupeds need studying [179,180,380]. Are there metabolic differences in GPs related to the anthropometric findings for girls [47-49], and in trunk width GPs of human babies compared with nonhuman primate babies? (see above, Evolutionary Origins).

(8) Evaluation of circulating hormones - leptin [12,163,164,206,207,381], high affinity leptin binding protein (soluble leptin receptor) [378,379], growth hormone [307,308], IGF-I and binding proteins [5,379], and estrogen levels [307,327-329] - in AIS girls by relatively higher and lower BMI subsets, with a view ultimately to a possible clinical trial of medical treatment by a somatostatin analogue [319] and β-blockers [199]. Cross-sectional [163,164] and longitudinal [12,381] studies are needed.

(8) Evaluation of receptors to hormones in growth plates and intervertebral discs including growth hormone, IGF-I, leptin, estrogens and melatonin by relatively higher and lower BMI subsets [180,220,222,223,382-384].

(10) In AIS spinal curves, correlation studies between MRI and histomorphology of spinal growth plates obtained at surgery [43-45] need extending.

(11) Sensory and sympathetic innervation of vertebral endplates in patients with idiopathic scoliosis needs more evaluation [385]. In this connection, sympathectomy as a possible prophylactic procedure for AIS in girls, and as a test of the LHS concept, needs consideration.

(12) Search for extra-spinal skeletal length asymmetries in AIS girls in other bilateral bones - sacral alae [153-155], clavicles and scapulae (Figure 1).

(13) Assessment of autonomic nervous system function in AIS girls [25,211,286,287,352,353,358,363,386,387]. In *lower BMI subset *AIS girls, is sympathoactivation stronger without any increase in GH/IGF secretion, and *vice versa *in *higher BMI subset *AIS girls?

(14) Estimates of body fat [386,388] including brown adipose tissue [205,299,389-395], BMI [161-171] and relation of the latter to calcium intake [396] and genetics [172-175] in AIS girls.

(15) The suggestion that the putative hypothalamic dysfunction of AIS in girls is enhanced by raised circulating leptin levels associated with fat accumulation of female puberty suggests that, where appropriate, lowering circulating leptin levels from BMI reduction may diminish scoliosis curve progression in some girls. In this connection, besides dieting, increasing calcium intake [396] and manipulating the function of brown adipose tissue [299,389-395] need consideration.

(16) As in the Rett syndrome [286,287] skin sympathetic responses need studying in AIS girls, separately for higher and lower BMIs, and subjects with the Prader-Willi syndrome, with the recording electrodes placed on both sides of the trunk and at other sites.

(17) The hypothalamus, neuropharmacology and neuropsychology, all need evaluation by neuroscientists in relation to *the LHS concept of the double neuro-osseous theory *particularly of a) negative regulators of leptin transduction, including SOCS-3 [243,244,250], PTP-1B [240,252], and OB-RGRP [247,248,253], and b) the positive regulator SH2B1 [232]

(18) Whether *SOCS-3*, *PTP-1B *and *SH2B1 *are significant contributors to AIS pathogenesis has to start with an examination of genetic association between phenotype and variation at each of these genes.

(19) According to Mattson [282], interventions that activate hormetic signaling pathways in neurons is a promising new approach for the prevention and treatment of a range of neurological disorders. *Hormesis *and the dose-response of leptin/bone growth in AIS girls [283-285] need more study [36] (Calabrese EJ, personal communication].

(20) The studies of girls with right thoracic AIS (Figure 6) need evaluating in girls with left thoracic and other types of AIS, and include hormonal and sympathoactivation comparisons.

(21) The above studies in girls, AIS and normals, need similar evaluation in boys [47-50,117-122] to establish gender similarities and differences [397]. Do adolescent boys with societally-increased fat accumulation have a raised prevalence of progressive AIS?

(22) *Infantile idiopathic scoliosis *(IIS, early onset scoliosis) occurs at the younger period of life when the human body is growing rapidly and both boys and girls accumulate fat transiently. Curve resolution/progression in boys and girls with IIS is established in relation to rib-vertebra angles [398,399]. The natural history of IIS, resolving and progressive, needs further study in relation to other variables including trunk widths, adipose tissue, and epidemiological findings that may be explained by the functions of white and brown adipose tissue (WAT and BAT). The variables are:

• the funnel-shaped upper chest in progressive IIS [400];

• *biacromial and biiliac widths *are narrow relative to *sub-ischial height *(SIH) in older IIS boys and girls (Figure 16), while SIH is not abnormal [401,402].

**Figure 16:**
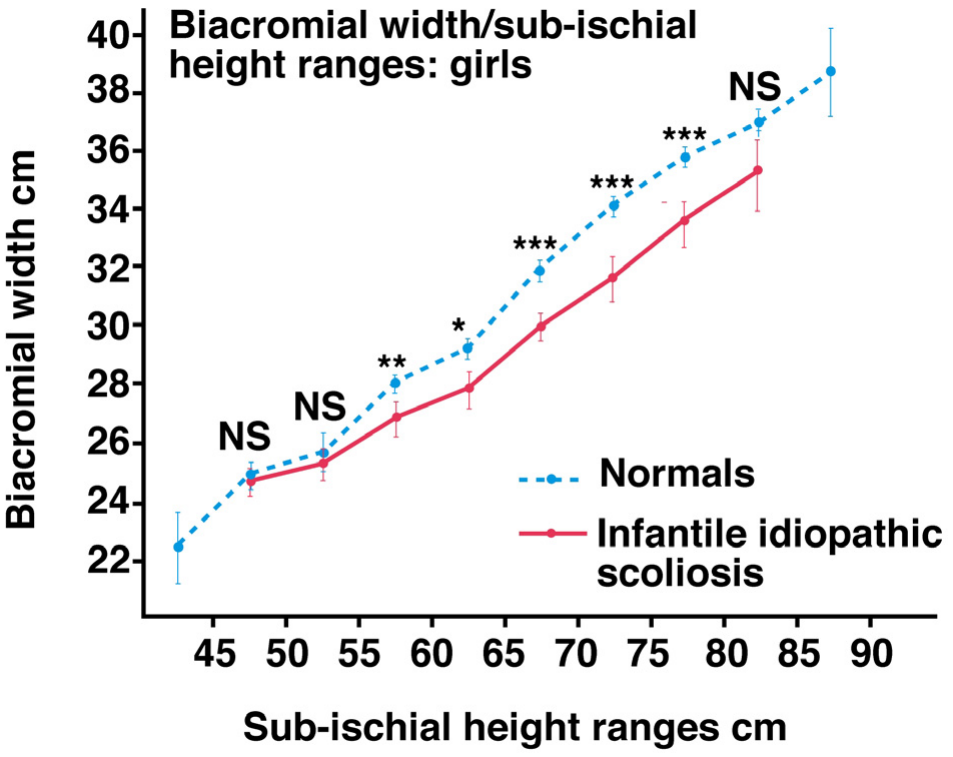
**Girls with infantile idiopathic scoliosis (IIS) and normal girls**. Biacromial width plotted against 5 cm ranges of sub-ischial height (SIH = standing height *minus *sitting height). The shoulders of the girls with IIS are significantly narrower relative to the normal girls at most of the SIH ranges above 55 cm (mixed longitudinal data by two observers (RGB & PHD) from subjects in Birmingham, Nottingham and Liverpool during 1972-76; n = 91, means ± 1 standard error, statistical significance levels for p values from t-test * = 1-5%, ** = 0.1-1%, *** p < 0.001, NS = not significant) (Diagram redrawn from Dangerfield et al [402]).

• in infants developing IIS under 6 months, there was an excess of curve onset in the two winter quarters and of premature low birth weight males [403];

• the declining prevalence of IIS [404] in lower socio-economic groups in the UK [403] in relation to a) the interscapular pad of BAT, its sympathetic innervation and non-shivering thermogenesis [389,391,395,401,405,406], and b) the central heating of homes over the period of study;

• the loss of subcutaneous fat in subjects with malignant progressive IIS about 4-6 years of age [407]; and

• in normal boys and girls, the dramatic decline from chubbimess to a comparably lean condition by 5 years of age with greater interscapular BAT in premature than mature infants [299-302].

Overall, these findings suggest the hypothesis that white and brown adipose tissue, leptin, hypothalamus and the sympathetic nervous system may, collectively, play a role in the pathogenesis of IIS.

(23) In addition to the historical reductionist approach, a systems-biology approach [408] is needed to evaluate the pathogenesis of AIS, as for obesity [301]. This approach involves multidisciplinary research leading to new theories and new experiments.

## Conclusion

(1) The *double neuro-osseous theory *for AIS pathogenesis in girls postulates developmental disharmony between autonomic and somatic nervous systems expressed in the spine and trunk and exaggerated by hormones producing systemic skeletal overgrowth (preoperative girls) (Figures 1 and 7).

(2) The theory predicates AIS pathogenesis in girls on dysfunction in one or both of two putative *normal *mechanisms involved in trunk growth, each acquired in evolution and unique to humans.

(3) The autonomic component of the *double neuro-osseous theory *for AIS pathogenesis in girls usually involves *selectively increased sensitivity *of the hypothalamus to the circulating adipokine leptin, with asymmetry routed bilaterally via the sympathetic nervous system to the growing axial skeleton where it initiates the scoliosis deformity. We speculate that increasing levels of circulating leptin [12] with the fat accumulation of adolescent girls [299,301], enhance the increased hypothalamic sensitivity to leptin.

(4) In the autonomic nervous system, the putative dysfunction - selectively increased hypothalamic sensitivity to leptin as *up-regulation *from mutation(s), *may be regulated by one or more of five possible molecular mechanisms*. The abnormal hypothalamic asymmetry is attributed to hormesis [36,124,282-284].

(5) In the *somatic nervous system*, dysfunction of a putative *postural escalator mechanism *involving the *central body schema *fails to control, or may induce the spinal deformity of AIS girls (*escalator concept*) (Figures 1 and 3).

(6) The developmental disharmony in the trunk is compounded by any relative osteopenia of vertebrae, biomechanical spinal growth modulation, accelerated disc degeneration, and platelet calmodulin dysfunction.

(7) Biomechanical factors acting during growth may localize thoracic AIS and contribute to its sagittal spinal shape alterations [83-90]; these include ribs [59-63] and/or vertebrae [64,65,91-93], and spinal cord [64,65].

(8) The hypothalamic dysfunction of the double neuro-osseous theory is expressed as:

• Sympathoactivation expressed asymmetrically in vertebral plates - left-right, front-back and/or torsionally - and in some paired bones.

• Increased hypothalamic sensitivity to circulating leptin (*up-regulation*) in some younger AIS girls with larger curves also involves the GH/IGF-I axis [222] (Figures 5, 7 and 9).

• Hormonal effects cause exaggeration of the sympathetic-induced vertebral/rib asymmetry(ies) contributing to progression of larger (preoperative) AIS curves in girls.

• Curve progression is postulated to involve an inverse relation of sympathoactivation and GH/IGF secretions (Figure 5). An inverse relation of these functions is found in several medical conditions.

(9) Progress towards these interpretations started in 2008, when theories were summarized which led us to propose a novel *neuro-osseous escalator concept *for AIS pathogenesis in girls affecting the somatic nervous system (Figures 1, 2 and 3) [51,111].

(10) Subsequently, anthropometric data from three groups of adolescent girls - preoperative AIS, screened for scoliosis and normals, were analysed by an original method for scoliosis of comparing data between subsets of relatively higher and lower body mass index (BMI).

(11) New findings revealed: *energy priority of trunk width growth (Figures *4*and *5) [46,117-119], *skeletal asymmetries *(Figure 6) [46,120,121], and *skeletal overgrowth patterns *for age (Figure 7) [29,122]. The contrasting skeletal features were not explained by any of the theories of AIS pathogenesis surveyed [51] including the *escalator concept *[51,111].

(12) The autonomic nervous system component of the theory (*LHS concept*) [25] draws evidence from several fields including:

• thoracospinal concept for the pathogenesis of right thoracic AIS in girls [59-63];

• new neuroskeletal biology relating the *sympathetic nervous system *to bone formation/resorption and bone growth [187-198];

• white adipose tissue, the adiposity hormone *leptin *secreted by adipose tissue which functions as a *sentinel *of energy balance and long-term adiposity to the *hypothalamus*; and

• central leptin resistance in obesity and possibly in healthy females.

(13) A new hypothesis for AIS pathogenesis in girls is formulated incorporating white adipose tissue, energy homeostasis (bioenergetics), the hypothalamus and sympathetic nervous system, in a disorder presenting as asymmetric abnormalities of trunk growth and, as suspected in preoperative girls, with systemic skeletal overgrowth.

(14) The endocrine and therapeutic implications of the *LHS concept *are discussed. An immediate need is to evaluate circulating hormone levels in AIS girls by relatively higher and lower BMI subsets; and later a possible clinical trial of medical treatment by a somatostatin analogue and β-blockers.

(15) Some methods for testing the theory's hypotheses are outlined.

(16) The putative hypothalamic dysfunction is thought to have an evolutionary origin in hominid fat deposition which in more than 3 million years, may have provided energy needed *sequentially *for each of:

• trunk width growth at the pelvis (mainly sacral alae), (Figures 5 and 12);

• trunk width growth of upper thorax and shoulders (Figures 10 and 11); and

• brain growth with

• pelvic depth increase (Figure 12).

We postulate that white adipose tissue still provides for skeletal growth processes in fetal and post-natal normal human development [299-302].

(17) In some normal juvenile girls, but not boys, the hypothalamus may function with *central (hypothalamic) leptin resistance of the somatotropic *(GH/IGF) *axis *to prevent too much energy being invested in female skeletal growth, thereby conserving energy for reproductive development. AIS is viewed as expressing central leptin *sensitivity *of hypothalamic sympathetic function and, in some younger preoperative girls, of the somatotropic neuroendocrine axis (Figure 7).

(18) A new interpretation involving the hypothalamus for some melatonin-deficient mouse models of scoliosis is presented.

(19) Evidence for *infantile idiopathic scoliosis *is outlined suggesting a need to evaluate the hypothesis that white and brown adipose tissue, leptin, hypothalamus and the sympathetic nervous system may play a role in its pathogenesis.

## Competing interests

The authors declare that they have no competing interests.

## Authors' contributions

RGB and RKA undertook the day-to-day research producing results which RGB interpreted theoretically in relation AIS pathogenesis in discussion with MPG, PHD, AM, TLR and SIA. MPG has expert clinical knowledge of scoliosis, PHD in human growth studies including scoliosis, AM in research relating to pediatric orthopedics, TLR expert clinical knowledge of pediatric endocrinology and diabetes, and SIA special knowledge of bone biology.

## Appendix 1

*Some theories of AIS pathogenesis *[51]

(1) Genetics [75-79].

(2) Biomechanical spinal growth modulation [80-82].

(3) Relative anterior spinal overgrowth (RASO) [83-90].

(4) Dorsal shear forces and axial rotation instability [75,91].

(5) Asynchronous spinal neuro-osseous growth [64,65,92,93].

(6) Postural abnormalities and hind brain dysfunction [69,94-103].

(7) Motor control problem [104].

(8) Body-spatial orientation concept [69].

(9) Neurodevelopmental concept [105,106].

(10) Thoracospinal concept [59-63].

(11) Systemic melatonin deficiency [7-9].

(12) Systemic melatonin-signaling pathway dysfunction [14-20].

(13) Systemic platelet calmodulin dysfunction [21,22,107].

(14) Symmetry control dysfunction - developmental instability [108-110].

(15) Collective and escalator models [51,111].

(16) Leptin-hypothalamic-sympathetic nervous system (*LHS*) dysfunction with disharmony between somatic and autonomic nervous systems in the spine and trunk [24-29].
